# Functional Copy-Number Alterations in Cancer

**DOI:** 10.1371/journal.pone.0003179

**Published:** 2008-09-11

**Authors:** Barry S. Taylor, Jordi Barretina, Nicholas D. Socci, Penelope DeCarolis, Marc Ladanyi, Matthew Meyerson, Samuel Singer, Chris Sander

**Affiliations:** 1 Computational Biology Center, Memorial Sloan-Kettering Cancer Center, New York, New York, United States of America; 2 Department of Physiology and Biophysics, Weill Cornell Graduate School of Medical Sciences, New York, New York, United States of America; 3 Department of Medical Oncology, Dana-Farber Cancer Institute, Boston, Massachusetts, United States of America; 4 Broad Institute of Harvard and Massachusetts Institute of Technology (M.I.T), Cambridge, Massachusetts, United States of America; 5 Sarcoma Biology Laboratory, Sarcoma Disease Management Program, Memorial Sloan-Kettering Cancer Center, New York, New York, United States of America; 6 Department of Pathology and Human Oncology and Pathogenesis Program, Memorial Sloan-Kettering Cancer Center, New York, New York, United States of America; 7 Department of Surgery, Memorial Sloan-Kettering Cancer Center, New York, New York, United States of America; The University of Queensland, Australia

## Abstract

Understanding the molecular basis of cancer requires characterization of its genetic defects. DNA microarray technologies can provide detailed raw data about chromosomal aberrations in tumor samples. Computational analysis is needed (1) to deduce from raw array data actual amplification or deletion events for chromosomal fragments and (2) to distinguish causal chromosomal alterations from functionally neutral ones. We present a comprehensive computational approach, RAE, designed to robustly map chromosomal alterations in tumor samples and assess their functional importance in cancer. To demonstrate the methodology, we experimentally profile copy number changes in a clinically aggressive subtype of soft-tissue sarcoma, pleomorphic liposarcoma, and computationally derive a portrait of candidate oncogenic alterations and their target genes. Many affected genes are known to be involved in sarcomagenesis; others are novel, including mediators of adipocyte differentiation, and may include valuable therapeutic targets. Taken together, we present a statistically robust methodology applicable to high-resolution genomic data to assess the extent and function of copy-number alterations in cancer.

## Introduction

Human cancer is caused in part by irreversible structural mutations. These can produce changes in DNA copy number at distinct locations in the genome [1]. Aberrations of this type affect the function of genes and thereby produce a transformed phenotype. Comprehensive characterization of these aberrations is a necessary step in understanding disease etiology and advancing the development of targeted therapies [2], [3], [4], [5], [6], [7]. Techniques based on microarray technologies can simultaneously measure thousands to millions of loci in the genome for DNA copy number changes. They include array comparative genomic hybridization (array CGH) and single-nucleotide polymorphism (SNP) arrays (reviewed in [8]). These increasingly sensitive technologies have been used to characterize not only aberrations in cancer, but also to describe copy-number variation in the human population [9], and the basis of genetic disorders (reviewed in [10]).

Given its capacity to identify novel oncogenes and tumor suppressor genes in cancer, two strategies have been used to analyze copy number array data from tumors. The traditional approach segments noisy probe-level data in individual tumors (dividing the genome into regions of equal copy number) [11], [12], detects aberrations with a global threshold, and heuristically defines boundaries of regions of frequent change [13], [14]. Newer algorithmic strategies use statistical models for the analysis of multiple samples [15], [16], [17]. More recently, Beroukhim et al. proposed an interesting comprehensive framework for assessing copy-number alteration in tumor cohorts [18]. In parallel to these computational developments, efforts are underway to analyze large tumor collections in a variety of cancer types, such as the pilot phase of The Cancer Genome Atlas [19] [The Cancer Genome Atlas (TCGA) Research Network 2008, submitted]. These will be collected using diverse sources and criteria that likely result in intra-tumor heterogeneity and between-tumor variability. Therefore, important unresolved issues remain. How should alterations in individual tumors be detected and combined when a collection of samples vary substantially in their noise characteristics? How should the genome be divided and assessed to more naturally reflect how alterations arise? What are the features of a realistic background model that allow for the identification of statistically significantly recurrent and therefore more likely functional alterations?

**Figure 1:**
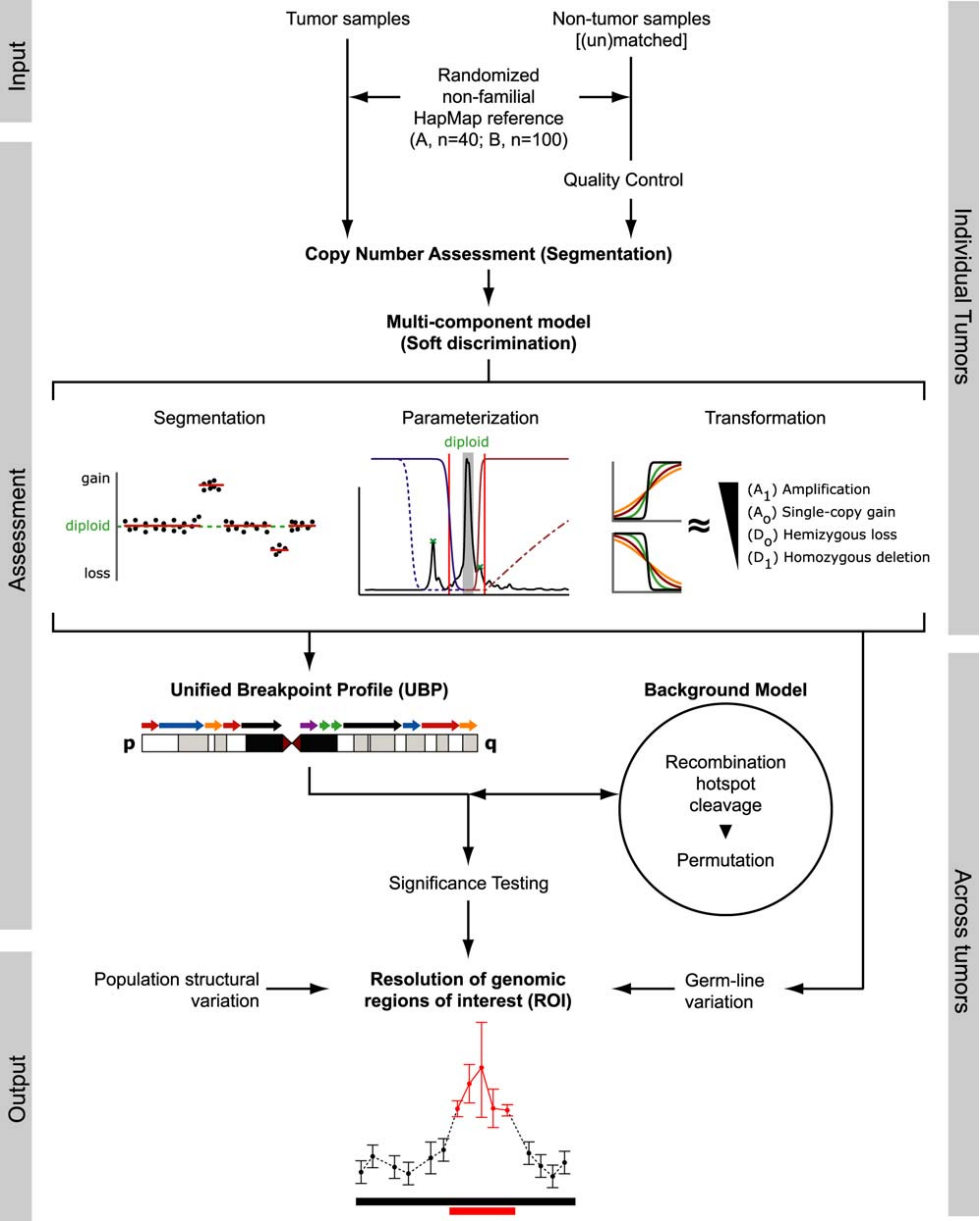
Overview of the RAE workflow. Input is a set of patients; tumor DNA, (un) matched non-tumor DNA, and an unrelated reference normal cohort. Tumor and non-tumor samples are quantified, normalized, and subject to quality control. In the assessment phase, individual samples are segmented and a multi-component model is parameterized for each; this produces a detector for single-copy gain, amplification, hemizygous loss, and homozygous deletion. Across all tumors, a unified breakpoint profile (UBP) is derived from the ensemble of segmentation breakpoints, and each region is scored for gain and loss. A background model of random aberrations is constructed with supplemental cleavage and permutation of genomic regions, and p-values are assigned and corrected for multiple hypothesis testing. In the output phase, RAE determines genomic boundaries for regions of interest (ROI), controls for germline and population copy-number variation, and reports statistically significant alterations.

In this article, we describe a computational framework that addresses each facet of this problem. We (i) develop distinct scoring models for different alteration types, with parameters adapted to the characteristics of individual tumors, (ii) use segmentation breakpoints to divide the genome for analysis that stresses the physical nature of copy-number alteration, (iii) build a random aberration model that approximates the biological process by which alterations arise, and use it to (iv) assess the statistical significance of observed alterations. This identifies genomic regions of interest (ROI) altered more frequently than would be expected by chance, and therefore more likely to drive tumorigenesis (Figure 1). We apply our method to a large repository of solid tumors to test its performance. We also apply RAE to a novel high-resolution copy number data set generated in our laboratories for a set of pleomorphic liposarcoma samples to illustrate its capacity to lead to novel discoveries.

## Results

### Extrinsic sources of variation

In the first phase of RAE, we address the issue of reliably detecting copy-number alteration in individual tumors. Every tumor, including those from patients with the same type of cancer, varies in their noise characteristics. We focus here on experimental noise and the problem of inhomogeneity of tumor DNA. An additional source of biological noise is structural variation, which we address later. Regarding the former, we found at least four distinct causes that can obscure copy-number changes in a tumor and this motivates our departure from global thresholds for detecting alterations. They include (i) low-quality matched non-tumor DNA samples, (ii) stromal admixture, (iii) tumor heterogeneity, and (iv) incoherent tumor profile, and we discuss each in turn.

#### Variation in quality of matched normal samples

Many groups, including our own, have observed significant non-diploid copy number in some normal samples (Figure S1). Causes may include the source tissue (in the case of *normal* tissue adjacent to tumor), differing handling protocols between tumor and normal samples, prior chemotherapy on DNA of normal blood cells, circulating tumor cells, and other contamination of normal DNA. In a *paired* analysis, this non-neutral signal will attenuate or otherwise alter the tumor's signal. To prevent this, we substitute a reference normal dataset of known diploid phenotype and analyze tumors in an unpaired format (Methods). This reference is generated by randomly selecting a subset of unrelated individuals of the HapMap collection, and produces a consistent diploid signal for tumor quantification and normalization (Methods S1, Table S1, and Figure S2). We further reduce noise in this new intensity ratio by segmenting individual tumors [11], [12]. This process correlates neighboring markers of common copy number, assigning the arithmetic mean of probe-level signal across the markers in each segment (Methods). While we avoid the use of matched normal DNA at this step, we do use a high-quality subset for germline event filtering after statistical assessment (Methods).

#### Stromal admixture

The second source of noise is tumor impurity, a well-documented problem [20], [21]. Individual tumors have different levels of non-tumor cell contamination. This reduces the ratio of signal-to-noise within and between tumors. It also compromises accurate genotyping for concurrent loss-of-heterozygosity (LOH) analyses. This jeopardizes the detection of two important classes of alteration: copy-neutral and deletion-associated LOH. Contamination of tumor DNA by non-neoplastic cell DNA exerts its effect globally, equally suppressing signal at all loci in a tumor. Our solution is two-fold. First, we take an individual-tumor approach to setting thresholds in log_2_ signal to detect aberrations, thereby extracting information from tumors that otherwise provide insufficient signal to detect non-diploid copy number changes in comparison to purer tumor samples. Second, we standardize the magnitude of alteration in all tumors to facilitate between tumor-comparability, an important feature when comparing tumors of varying stromal admixture.

#### Tumor heterogeneity

The third source of noise is perhaps the most confounding. We see evidence of an intermediate copy number in multiple tumor types. For example, when the value of a monosomy (or ChrX in a male patient) establishes with confidence the continuous log_2_ value corresponding to discrete integer copy loss, this signal is often an arm-length loss that falls halfway between diploid and the log_2_ value of single-copy loss. This may be allele-specific copy number exclusive to either the maternal or paternal chromosome, or more likely indicates the possibility that multiple distinct but related subclones exist within a single clonal tumor. When single-copy loss of a chromosome exists in only one of two distinct tumor cell populations, there is a convolving of alteration, reducing the magnitude of the event when measured from the mixed population (Figure S3). Therefore, multiple putative tumor cell populations differentially affect signal in a *local* manner, at distinct regions in the same tumor. Consequently, we chose an individual-tumor alternative to a global threshold for alteration, the former being more sensitive to the detection of this sort of cryptic signal.

#### Incoherence of copy-number profile

Finally, inaccuracy in copy-number segmentation is the last extrinsic source of variation compromising event detection in individual tumors. A large amount of information is encoded by original probe-level data on dense arrays such as the Affymetrix 250 K SNP array. Segmentation is designed to reduce that information content to a minimal set of discrete gains, losses, and neutral copy number. The greatest reduction in information is in samples producing few segments, and least in samples of high segment count (Figure S4). However, this does not have a coherent relationship to probe-level noise (Eq. 1, Methods). Consequently, because the features of probe-level noise are different from those of segmentation, we use only the latter at all subsequent stages of analysis.

### Multi-component scoring model for copy-number alteration

To adapt to this diversity of variation among individual tumors, we developed an adjustable multi-component model to detect aberrations, the first core feature of RAE. We begin by separating segmented copy-number into four *components*, each encoding the status of an alteration type; single-copy gain (A_0_), amplification (A_1_), hemizygous loss (D_0_), and homozygous deletion (D_1_). This separates both the analysis of total gain from loss, but also specific and intuitive classes of each. This is necessary because each alteration presents different analytical challenges, not only in dynamic range, but also in their noise characteristics, which is often overlooked. Also, by dividing total signal into these four distinct classes, it is possible the model can extract more information and produce higher accuracy in individual event calls.

#### Gain

In the analysis of a set of tumors, there are two attributes that describe copy-number gain, frequency and amplitude. At the single-sample level, this equates to a “detector” and an “integrator”, the former identifying the existence of an event and the latter assigning it a magnitude proportional to its original amplitude. We reasoned that encoding the detection of an event separately from its amplitude would have several benefits: (i) a detector operates at the margins of signal and noise and must be robust to the introduction of wild-type signal, (ii) because amplitude is unbounded and varies as a function of stromal contamination, it should be standardized to facilitate between-tumor comparability, and (iii) in our statistical model that tests whether an alteration exceeds a random aberration rate, which is based primarily on recurrence across samples, we want to boost our power for detecting infrequent but very high-amplitude events. So, these are separately encoded as single-copy gain (A_0_) and amplification (A_1_).

#### Loss

We approach the analysis of genomic loss slightly differently, though with a similar conceptual framework. There are several challenges unique to allelic loss that justifies a modified approach, and each of these has an important biological corollary. First, deletion is restricted in its range; only two copies of a locus can be lost. This is different than amplification. Lacking real magnitude, DNA is either “present” or “absent”, and therefore an identical scoring scheme would be inappropriate. This complete absence of signal (or magnitude) corresponds to homozygous deletion. The second analytical complication is negative skew in the distribution of segmentation around the diploid peak (Figure S5). Thus far, this is a feature unique to genomic loss and complicates the detection of hemizygous loss when its transition from wild-type signal appears featureless. Nevertheless, accurately detecting single-copy loss is important. The biological parallel is a classical tumor suppressor model, one in which somatic mutation or methylation in one allele is coupled to loss of the other. These losses are often broad, and may target multiple loci, reducing the function of more than one gene. However, this falls at the margins of detectability in such a noisy system. To overcome these complexities, we also separate deletion into two components. Unlike the model for gain, both components are “detectors”, one for hemizygous loss (D_0_), and the other for homozygous deletion (D_1_) (parameterization discussed in Methods S1).

#### Soft discrimination

**Figure 2:**
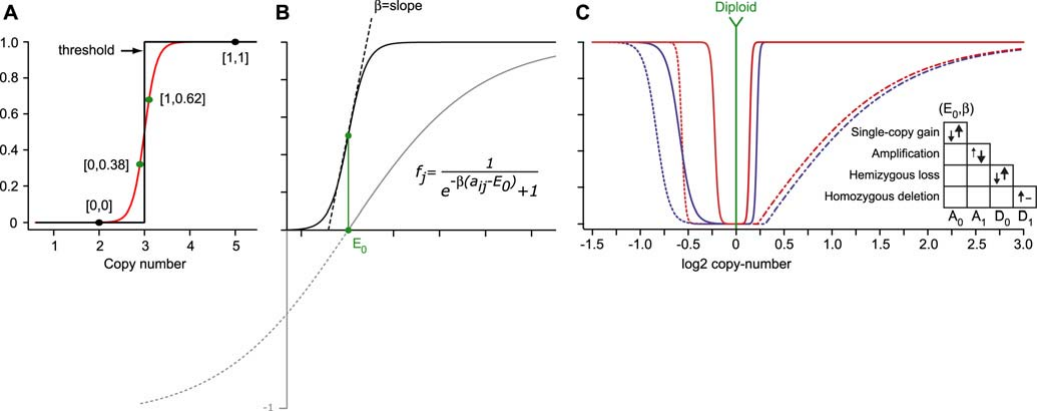
Multi-component model of copy-number alteration. (a) In a noisy system, a soft discriminator (red) is juxtaposed to a hard threshold (black); both of which assign points either continuous or binary values respectively (parentheses) for confidently copy-neutral or amplified loci (black) and for challenging cases at the margin of signal (green). This indicates the benefit of soft discrimination. (b) The functional form of the soft discriminator; a sigmoid function with parameters for location (*E*) and slope (*β*). (c) Individual-tumor approach to detecting gain and loss; the multi-component model parameterized for two tumors (red and blue) indicating that tumor-specific features produce different discriminators for single-copy gain and loss (solid), amplification (dot-dash), and homozygous deletion (dotted). Parameterization selects values for *E* and *β* such that their magnitude (unsigned) moves in the direction indicated (legend).

While there are many options for detecting these alteration types, a key feature of our approach is the use of *soft* discrimination. Providing a robust (and binary) value for the existence of an event in a noisy system is difficult. This is exacerbated for single-copy events at the margins of signal and noise. Consequently, we found that even after segmentation, a dataset-wide log_2_ threshold for detecting alteration underperforms in such a noisy system (data not shown). Alternatively, there is significant precedent for using soft discriminators in noisy systems, and we adapt this principle to detect copy-number alteration. For example, consider alteration of a locus in two tumors, both having similar amplitudes. The former exceeds a *hard* threshold by a small magnitude; the latter does not, but again by only a small magnitude. It is unlikely that this nominally similar locus results in altered biology in the former, but the latter is effectively penalized (Figure 2A). So, to achieve soft discrimination of each alteration type, we use a sigmoid function with parameters for location (*E*) and slope (*β*) (Figure 2B, Methods). This function maps continuous log_2_ ratios, theoretically spanning ±∞, to a constant value between 0 and ±1 (depending on the sign of *β*). By varying the magnitude of *β*, we can make the function behave more or less like a sharp threshold. Additionally, because the parameters (*E*,*β*) are determined from individual tumor data and adapted to each alteration type, we can vary the function's sensitivity, accommodating the very different patterns of noise previously discussed (Figure 2C, Methods S1). This adaptive parameterization is also a mechanism by which we can extract information from even the most challenging tumor profiles. This flexibility partially eliminates the need for subjective quality control in the elimination of fundamentally uninformative samples. For individual tumors having a complex and/or incoherent pattern of signal (Figure S5), parameterization produces conservative values of *E* and *β* for each alteration type, suppressing a large fraction of the total signal by design. This is especially important for the analysis of uncommon tumor types where source material is at a premium and the elimination of samples a distinct drawback. Finally, when soft discriminators for single-copy gain and for mono- and biallelic losses are combined across all tumors, they are a proxy for the recurrence of each alteration type. This aggregation across tumors is the subject of the next section.

### Aggregating alterations

#### A unified breakpoint profile (UBP)

**Figure 3:**
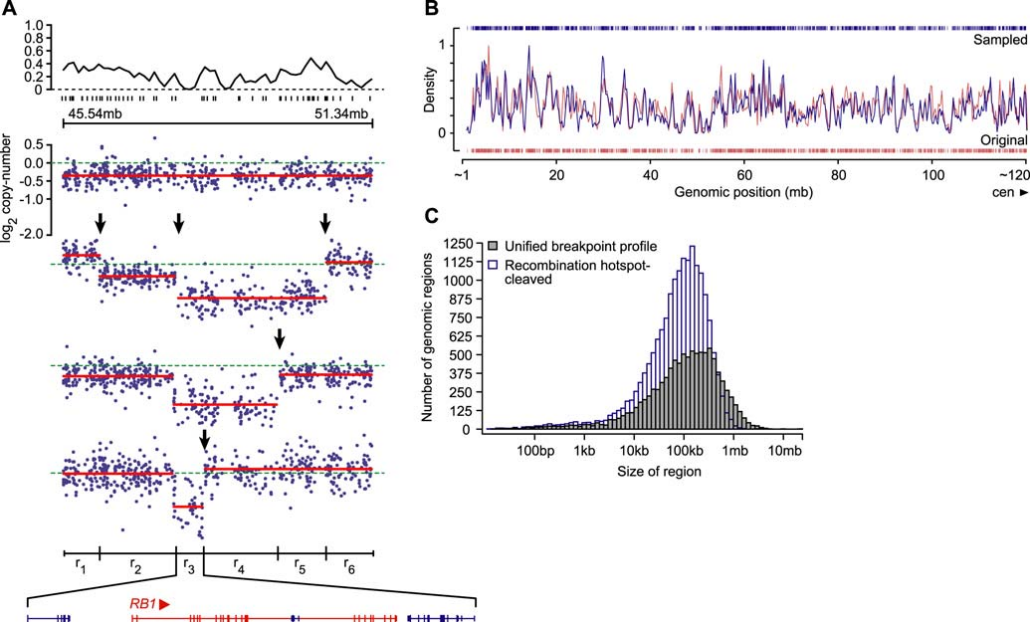
Aggregation and permutation. (a) The density of human recombination hotspots (top; median distance between hotspots is ∼55 kb) spans segmentation (red) of probe-level data (dark blue) in a ∼5 mb region of 13q14.13-3 in four pleomorphic liposarcomas. The unique tumor-associated breakpoints (black arrows) define the UBP (regions r_1–6_; bottom), the smallest of which (r_3_) spans four genes including the tumor suppressor *RB1* (direction of transcription indicated). (b) On chromosome 1p, the density distribution of predicted recombination hotspots (red) at a width equal to the median distance between all p-arm hotspots (56 kb), and the distribution of their randomization (blue). The sampling procedure respects the shape of the original distribution and therefore the sequence features that underlie it. (c) Size distribution of regions derived from segmentation and subsequently defined by the unified breakpoint profile (UBP; gray), and those hotspot-cleaved regions of the same permuted during null model generation (as indicated, blue).

We were interested in identifying the most realistic unit of the genome on which alterations likely arise and for which our multi-component model should be assessed statistically. As with benign variants, pathogenic changes are segmental, altering ∼kilobase to whole-chromosome-sized stretches of DNA. Why analyze the data by evaluating a very dense set of markers (>238,000) when perhaps only 50∼20,000 are truly independent observations? Because lesions alter fragments of DNA, we felt RAE should operate on these. Therefore, we took advantage of the breakpoints produced by individual-tumor segmentation. This explicitly correlates neighboring probes on a segment with similar copy-number and approximates structural changes in the genome. We unify the unique breakpoint positions observed in all tumors and these create a new division of the genome (Figure 3A, Methods). These newly defined regions are cancer-type specific and the final unit of analysis. This avoids both an artificial length scale and the statistical compromises necessary when operating on individual markers, such as the impact on multiple-hypothesis testing when measurements are partially dependent (Methods S1).

#### Combining evidence of alteration from different tumors

To report a summary of alterations in these regions for a collection of tumors, we combined the detected alterations across all patients. The way in which we do this allows us to assess the significance of an event through comparison to a null distribution of purely random aberrations. Each component is first summarized as the average across samples in each region of the UBP. We then calculate a summary score (Eq. 3) for both total gain and loss (A′ and D′ respectively) that combines the evidence of the individual alteration types (Methods). The principal benefit of this approach is flexibility. A null model (the subject of the next section) can be created to evaluate: any combination of the original four components, summary scores for total gain and loss (default), or by weighting one alteration type relative to another. As a final aggregation step, we analytically derive uncertainty in this summary score for each region of the UBP. This is an important feature of our approach. By propagating the error of segmentation from all tumors spanning a given locus, we produce a representation of the uncertainty in our measurement of alteration at each locus (Methods S1). This uncertainty is an intrinsic feature of any scoring model, but is currently not used in existing methodologies.

#### A background model

We develop a background model for assessing the significance of tumor-specific alterations, the third core feature of RAE. The characteristics of a realistic background aberration model in human cancers are complex and an unresolved area of research. In a first approximation, we assume a tumor's profile is the combination of both driver and passenger alterations. Furthermore, regions selected by the tumor span genes whose perturbed function alters the normal cellular phenotype. We assume these are embedded amid non-specific aneuploidy, perhaps the product of increasing genomic instability. This fixes stochastically acquired changes during neoplastic progression, but which are fundamentally neutral to tumor biology. This suggests a process spanning the indiscriminate to the decidedly non-random, as well as a relationship between normal genetic turnover and the acquisition of copy-number change. This implies tumor-associated breakpoints identified by segmentation are only a small fraction of total breakpoints in the genome. So, we hypothesized that a background model should incorporate components of this benign genetic background. In the context of copy-number aberrations, we chose predicted human recombination hotspots.

Hotspots, a local increase in the rate of human recombination, are a feature of allelic and non-allelic ((N)AHR) homologous recombination. NAHR, in turn, is one mechanism by which *de novo* structural variants are fixed in the genome. A subset of these variants produces copy-number change, little of which is pathogenic. In fact, previous studies associate high rates of NAHR with segmental duplications. These sequences are therefore susceptible to break and rearrangement (reviewed in [22], [23], [24]). Moreover, copy-number variation is tightly coupled to segmental duplications in the human genome [9], [25]. Consequently, we use a random process involving recombination hotspots as a proxy for this mechanism. These hotspots are estimated from patterns of linkage disequilibrium (LD) between extant individuals, reflecting recombination occurring throughout their ancestral lineage [26]. We supplement tumor breakpoints in a manner consistent with both this higher-order structure of the human genome and patterns of genetic diversity.

We randomized the genomic positions of predicted recombination hotspots (*n* = 32,996, HapMap phase II [27]) with a rejection-sampling procedure that simulates the preferential features underlying the distribution of human recombination (Figure 3B). These randomized positions are used as cleavage sites for the largest tumor segments prior to permutation (Methods). Supplemental partitioning of the genome in addition to that provided by tumor segmentation prior to permutation also has an operational benefit. It increases the permutation space in a tumor when segmentation produces a low segment count of which a fraction are copy-altered, and the balance are large in genomic size but fundamentally diploid. Without additional division, the altered segment can be permuted into a finite number of positions, constraining the model. Fracturing the largest copy-neutral segments, however, provides a far greater count of positions into which the region of interest may be permuted.

Having investigated multiple permutation models, we chose a null distribution derived from genome-wide permutation (Methods S1). Briefly, (i) segments in each tumor are further subdivided (cleaved) at the positions of randomized recombination hotspots, after which (ii) the UBP is derived again on this modified ensemble of breakpoints (Figure 3C), (iii) the values of the multi-component model in each region of this UBP (A_0_, A_1_, D_0_, D_1_) are permuted together to another position of the UBP in each sample and re-combined across tumors (see Methods). This is typically repeated 10,000 times producing a null distribution of >10^8^ scored regions.

### Assessing significance and identifying regions of interest

To assign statistical significance, separately for gain and loss, we use this null distribution of permuted data to calculate p-values based on how often the randomly permuted score exceeds the sample score (Eq. 3). We then correct for multiple hypothesis testing with the Benjamini-Hochberg false discovery rate procedure [28]. This correction is done over all tests, which correspond to regions of the UBP. Depending on the segmentation profile of samples in a disease type, this results in a reduction of between one and three orders of magnitude in effective tests as compared to individual markers. The resulting q-value defines the fraction of tolerated false positives above a given score arising by random chance in our background model. Regions are then filtered based on the q-value with a typical cutoff of 0.01 (FDR≤1%).

#### Regions of interest (ROI)

**Figure 4:**
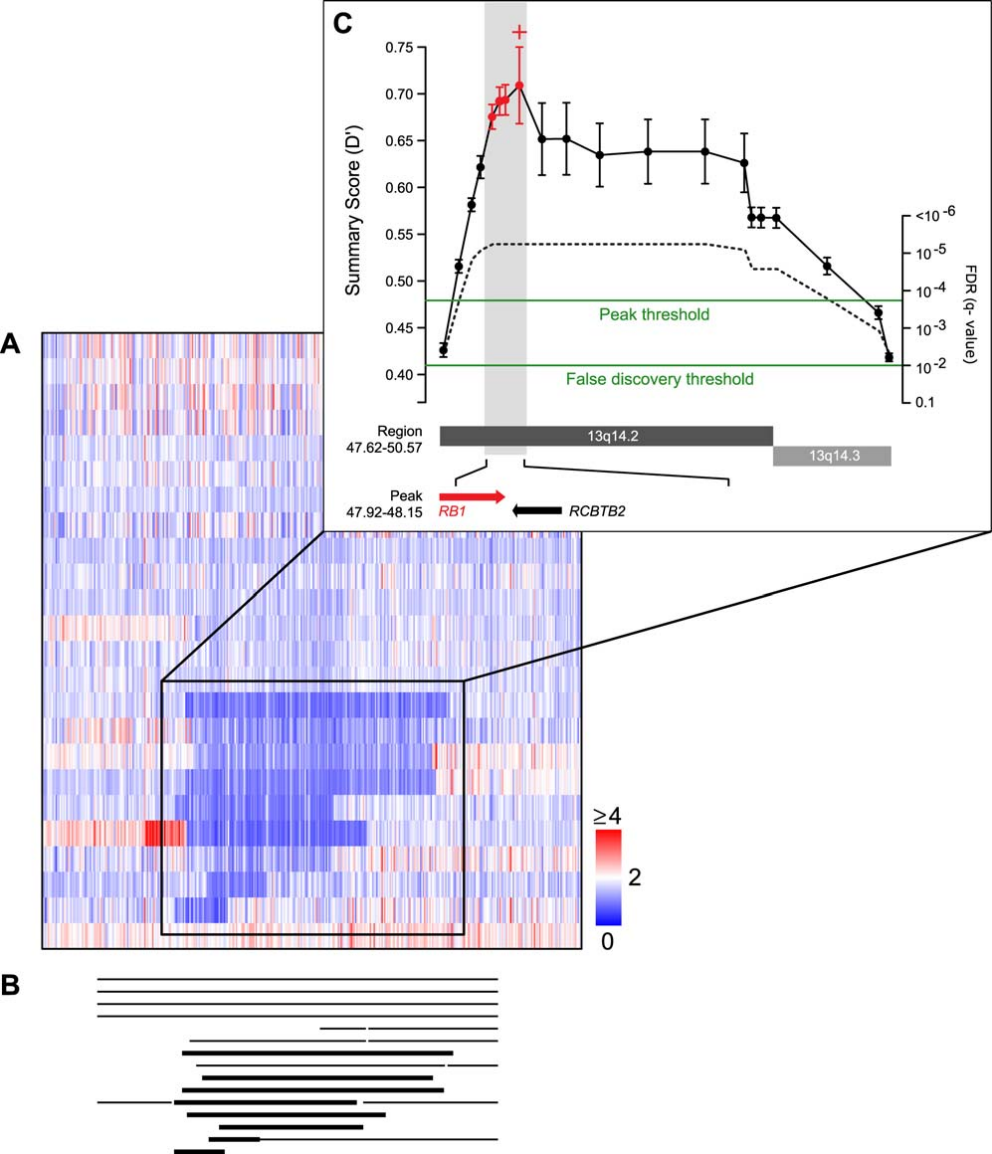
Regions of interest (ROI). Deletion of *RB1* at 13q14.2–q14.3 in pleomorphic liposarcoma demonstrates features of ROI detection in RAE. (a) Heatmap of copy number in a small region of 13q in 24 pleomorphic liposarcomas (tumors are rows, markers are columns; color scale as indicated), and (b) From segmentation, the extent of genomic deletion in a subset of tumors with either hemizygous loss (thin) or homozygous deletion (thick) (c) Inset, the regions of the UBP at this locus (filled circles), and their summary score (D′, left axis). The combination of analytical error (error bars) and two thresholds (FDR and peak detection, green) determine the sensitivity of ROI detection. The detected peak (identified by red plus) is merged with physically adjacent regions that fall inside its error interval (red filled circles and error bars) and define the 5 and 3′ boundaries of the ROI (gray). Statistical significance (q-value) corresponding to summary scores such that permutation is unable to resolve a p-value smaller than 1/(N_p_+1) (dotted line, right axis) indicates the necessity for resolving ROIs in the space of the summary score D′. Regional and peak boundaries define the ROI (at bottom; mb) spanning 20 and two genes respectively, the latter including *RB1* (direction of transcription indicated). Note, the region detected as the peak is void of genic content, emphasizing the necessity for incorporating a measure of uncertainty on its score.

We next explore the final core feature of RAE, determining the boundaries for regions of significant amplification and deletion. If an alteration contributes to oncogenesis, then we assume that region of the genome is selected for its effect on gene content. This event may alter a single gene or multiple independent events may target a coordinated program of genes. These lesions may also co-evolve with random alterations that have little biological impact. Non-random alterations are statistically significant relative to our null model and therefore are candidate regions of interest. Nevertheless, regions of interest are not rigorously defined, but are intuitive and motivated primarily by two issues. First, the biological researcher is interested chiefly in manageable and interpretable events, perhaps involving a single gene. Second, we see visually in the data regions of focality where peaks of alteration exist but are confounded by noisy data, including adjacent or neighboring peaks. To capture both of these, we implement a two-stage approach to determine ROIs. The first stage identifies regions of significant alteration (q≤0.01). These will be (i) isolated regions of the UBP (singletons) where focal alteration affects a single locus, or (ii) multiple physically adjacent regions that are merged and assigned the largest genomic boundaries of the event. The second stage is designed to interrogate these broad gains and losses for peaks of finer-scale and more significant alteration. These are more likely to contain oncogenes and tumor suppressor genes, meet the first intuitive criteria of ROIs, but are complicated most by the second. Consequently, there are two types of imprecision that affect the determination of regions of focal alteration. Spatial imprecision is related to the experimental system, where the *true* position of alteration is unmeasured due to marker selection, array composition, and finite resolution. Measurement imprecision refers to the error propagated from individual events in each sample and reflects both noise inherent in the experiment and the variability produced by sample size. The former is fixed and will improve as array density increases. The latter is something we incorporate explicitly into the second stage of our algorithm, but is missing from prior approaches [13], [14], [18], [29]. For a given broad region that includes loci exceeding a sensitivity threshold, we detect peaks in the summary score (L_2_, Eq. 3). If a peak is detected, it is merged with adjacent loci in this wider region of significance if their L_2_ falls within the peak's interval of error (Figure 4, see Methods). In this graphical representation from data, the *RB1* tumor suppressor, discussed in greater detail below, is detected in a peak of similarly merged regions that refines the boundaries of an ROI from those spanning ∼3 mb of sequence and 20 genes to those ∼237 kb spanning just two genes.

Using these intrinsic errors in the second-stage algorithm is important for several reasons. First, selecting only the maximally significant locus without consideration for adjacent significance in either the 5 or 3′ direction may produce artificially small peak boundaries. These may subsequently miss a gene proximal to, but not encompassed by the event (Figure 4). This potentially erroneous ‘maximum-peak’ identification may be caused by nothing more than artifact somewhere upstream in normalization, segmentation, or scoring. Second, and again an issue important to the analysis of uncommon tumor types, is the question of peak detection and target identification from copy-number alteration when the analysis is based on small sample sizes (∼20–50). There is a term in the measurement of error that scales as one over the square root of the sample size (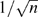). The difference in effect on error is acute between samples sizes available for rare tumor types versus those common epithelial tumor types.

#### Control for normal polymorphism in the human genome

The final phase of analysis is to control for observed germline and population copy-number variation (CNV). This is a common step in many studies. The assumption is that altered loci spanning observed variations in the genome should be removed given their ambiguity of origin, being either somatic possibly oncogenic or germline polymorphic. Nevertheless, three concerns arise. First, there has been little effort to interpret overlapping events, the biological significance of which is unclear when a variant's frequency in the population is low or undetermined. Second, existing CNVs are more accurately described as copy-number-variable regions, the exact boundaries of which are mostly unknown and perhaps over-estimated [30]. The third relates to specificity issues during CNV detection given the coverage of the sum of reported CNV. We implement a repository of approximately 10,000 autosomal variants (Methods). These span 745 mb of sequence and therefore the likelihood of CNV overlapping cancer-specific loci in a highly aberrant tumor type are high.

Our approach is conservative and controls for false negatives. Copy-number variants are analyzed for overlap with the focal event boundaries produced by RAE. We use the latter because aberrations tend to be larger in median genomic size than polymorphic events (data not shown). The percent sequence coverage is reported, and the event is classified as potentially polymorphic, but is not excluded at any coverage threshold. These simple criteria were designed to control for the removal of truly somatic events, such that a *de novo* duplication observed in a prior study would not invalidate a tumor-associated deletion at the same locus. A similar approach is used to screen cohort-specific germline variation (see Methods). However, because these appear in patient-matched non-tumor DNA, and may be interpreted in many ways, they are removed from primary results, but reported.

### Comparison in a large solid-tumor compendium

To assess the performance of RAE, we compared its results with those of a recently developed method on two large studies totaling 512 solid tumors (Methods S1). These included copy number array data from 371 lung adenocarcinomas and 141 primary and secondary gliomas [18], [31]. We found RAE produced good concordance with published focal events identified by the GISTIC method. In particular, RAE identified 29 of the 31 (94%) reported focal amplifications and deletions in lung adenocarcinomas, and 19 of 27 (70%) in glioma (Methods S1).

While we did not expect perfect agreement between the results of the two methods given their dissimilar analytic approaches, we investigated the differences in some detail. In the lung adenocarcinoma dataset, we quantified concordance in two different ways. To each region of the UBP derived by RAE for the lung dataset, we mapped published amplifications and deletions. We then compared amplified, diploid, and deleted regions between the two methods and found them to be highly concordant (χ^2^ statistic >10^4^, Methods S1, Table S4). We then assessed the relationship between each method's score (L_2_ in RAE and G-score in GISTIC) for reach region of the genome. In the regions of statistical significance from either method (FDR≤0.25 in both), we calculated the non-parametric Kendall tau rank correlation coefficient between summary scores of amplification and deletion. We found this reaffirms the high concordance (τ = 0.86 and 0.77 for amplification and deletion respectively). We repeated the latter concordance estimate on the glioma dataset and found very similar results (τ = 0.77 and 0.84 for amplification and deletion respectively; see Methods S1 for details). Additionally, we manually reviewed each locus reported by the original studies but not statistically significant by RAE (Methods S1). Overall, many subtle differences impact detection, and we describe two. First, the method for scaling tumor segmentation to a common baseline value (usually log_2_ = 0) affects the status of alterations in a small fraction of the most complex tumors (Figure S9). This is because common summary statistics like the median bears little relationship to the putative diploid peak in tumors where the latter is ill defined. The second affect is of greater impact. In the majority of tumors, the adaptive parameterization of *E_k_* from individual noise features is more stringent than are published symmetric thresholds in log_2_ copy number (Table S5, Figure S10). This suppresses possible signal that falls between the value of a symmetric threshold and *E_k_* in a given tumor. This produces a global reduction in frequency and therefore affects statistical assessment in kind. For events detected by another method and not RAE, it remains to be determined if these are truly real or the more stringent *E_k_* correctly suppresses these as false signal in the noisiest of tumors. In summary, we believe the regions of both agreement and disagreement highlight the value of investigating genomic data of this type with multiple approaches, leading to improved analysis methods and an increasingly complete and accurately derived profile of chromosomal aberrations [The Cancer Genome Atlas (TCGA) Research Network 2008, submitted].

### Application of RAE to Pleomorphic Liposarcoma

We applied RAE to a new dataset for an uncommon and challenging subtype of adult soft-tissue sarcoma. Soft-tissue sarcomas (STS) represent ∼1% of adult malignancies, yet their histological diversity, frequent presentation with advanced disease, and lack of response to conventional post-surgical treatment drives a total disease-specific mortality of 50% [32]. Of these, liposarcoma is the most common, accounting for 20% of all adult sarcoma. Liposarcomas are classified into three biological groups encompassing five subtypes, (1) well-differentiated/dedifferentiated, (2) myxoid/round cell, and (3) pleomorphic, based on morphological features and cytogenetic aberrations [33], [34]. Dedifferentiated and pleomorphic liposarcomas are characterized by complex karyotypes and have gross chromosomal aberrations [35], [36]. In contrast, myxoid/round cell liposarcomas have simple karyotypes with specific reciprocal translocations. We focus here on the most biologically aggressive subtype, pleomorphic liposarcoma.

Pleomorphic liposarcoma (pLPS) accounts for ∼8% of all liposarcomas, and represents only 1.6% of all soft tissue sarcomas. Nevertheless, they are highly undifferentiated tumors, are frequently located in the extremities, and have a disease specific survival of 60% at 5 years for patients presenting with localized disease [37]. Based on morphology alone, dedifferentiated and pleomorphic liposarcomas can sometimes be difficult to discriminate, even for the experienced soft-tissue pathologist. This distinction is important since patients with pleomorphic liposarcoma have a 3-fold greater risk of distant metastasis compared to patients with dedifferentiated liposarcoma. Previous work by our group identified a gene expression classifier that discriminates dedifferentiated and pleomorphic subtypes, but whose genes mainly reflect a complex amplification of 12q13-15 in the former [38].

**Figure 5:**
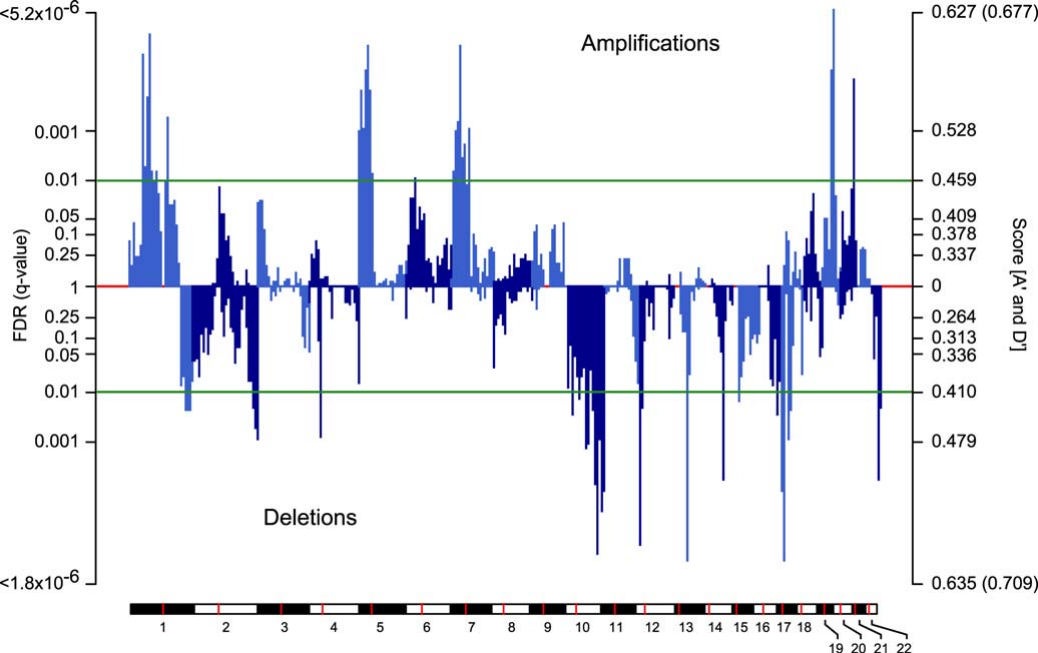
Statistically significant genomic alteration in pleomorphic liposarcoma. The false discovery rate (q-value, left axis) and score (A′ and D′, right axis) for amplification and deletion (positive and negative respectively, labeled) on the 22 autosomes in genomic coordinates (chromosomes indicated at bottom and in plot by alternating colors, centromere in red). The threshold for significance determines the alterations subject to ROI detection (green). Maximum observed scores of A′ and D′ unattainable by permutation p-value (parentheses, right axis).

A collection of tumor and non-tumor DNA specimens from 24 patients were selected and analyzed (Methods). A genome-wide perspective reveals pleomorphic liposarcoma has multiple regions of significant copy-number amplification and deletion (Figure 5). Nevertheless, whole-chromosome arm events, a feature characteristic of multiple epithelial tumor types, are only infrequently seen. RAE identified broad and focal alterations on 18 chromosome arms (Table 1, Tables S2–S3). On a fine-scale, several genomic deletions contain well-studied tumor suppressors, confirming prior observations in multiple karyotypically complex sarcoma subtypes [39]. This includes the most common genomic alteration, a deletion of 13q14.2–q14.3 including the *RB1* tumor suppressor (∼60% of tumors). This alteration is a mixture of hemizygous loss and less frequent homozygous deletion, the latter in five samples. *RB1* germline mutations in individuals with hereditary or bilateral retinoblastoma are associated with an increased risk of sarcoma development later in life [40]. Upon review, however, none of our pleomorphic liposarcoma patients had a history of retinoblastoma or prior radiation exposure.

**Table 1:** Alterations in the pleomorphic liposarcoma genome.

Locus	Region (Peak)*	Q-value†	Number of genes‡	Genetic elements of interest	Spanning known structural variation (CNV)§ #		
					Gain	Loss	Unknown
***Gain***							
1p31.2–p31.1	68313202–77461343	1.51E-05	27(1)	*TNNI3K,MSH4,hsa-mir-186*	8.8 (8.7)	11.1 (6.1)	6.9
5p15.33–p15.32	165712–5444231	1.86E-04	28	*TERT*	53.1 (22.2)	18.9 (5.9)	27.3
5p15.2	10731281–11126282	4.69E-03	2	*DAP*	-	38.1 (3.5)	-
5p15.2–p15.1	11413893–15762298∥	1.02E-03	7	*CTNND2,FBXL7*	3.7 (4.5)	7.7 (6.6)	14.1
5p13.3–p12	32750418–45758751	1.51E-04	64	*AMACR,C1QTNF3,RAD1,SKP2,LIFR*	8.5 (4.1)	4.1 (1.4)	2.3
7p21.3–p21.1	8732462–20510540	7.78E-04	26	*ETV1,TWIST1,TWISTNB,ITGB8*	21.1 (1.6)	29.5 (1.6)	14.5
7p15.3–p14.3	23715756–32063427	2.50E-05	51	*HOXA9,HOXA11,HOXA13,JAZF1*	8.8 (4.3)	6.7 (1.6)	5.5
19p12–q13.11ˆ	24161928–40254153	<5.22E-06	43	*CCNE1,LRP3,CEBPA*	7.3 (1.7)	3.5 (3.2)	11
	32690406–34776985	1.51E-05	1	*UQCRFS1*	4.7 (3)	16.2 (2.2)	27.9
	38552698–38678914	<5.22E-06	2	*CEBPG*	87.6 (2.5)	-	100
19q13.12	40680588–41040430	5.49E-03	21	*ETV2,MLL4,PSENEN*	-	3.7 (1.7)	-
***Loss***							
1q41–q42.12	216302655–221524000	4.51E-03	29	*MARK1,DISP1,TP53BP2,MIA3*	0.6 (1)	0.3 (1)	13.8
10q21.3–q22.1$	68571354–70982955	2.65E-05	24	*CXXC6,CCAR1*	3.8 (5.5)	3.1 (1.6)	7.2
10q22.1–q22.2$	72407412–76912067	2.65E-05	46	*MYST4*	14.8 (3)	1.4 (1)	4
10q24.32–q24.33	103687999–105303029	8.21E-06	33	*SUFU,NFKB2*	-	1.2 (1)	-
10q26.11–q26.3	119097873–135323432	5.35E-05	98	*CTBP2,FGFR2*	17.8 (4.6)	10 (5.1)	13.9
12p13.33	456768–1673782	1.15E-05	9	*RAD52,ERC1,WNT5B,ADIPOR2*	39.3 (4)	0.7 (1)	33.2
13q14.2	47917390–48154504	<5.74E-06	2	*RB1*	35.9 (1)	35.9 (1)	-
16q22.1	67103973–69257715	3.69E-03	32	*CTF8,CDH1,CDH3,FUK,NQO1*	14.3 (5.2)	31.1 (2.8)	21.3
17p13.1	6791092–7741807	<5.74E-06	57	*TP53* &	-	-	-
17q11.2	24079567–27921023	1.18E-03	49	*NF1,SUZ12*	5.1 (1)	4.7 (2.3)	8.2
22q13.1–q13.31	39296203–46069760	2.02E-04	84	*MKL1,ST13,EP300,XRCC6,PPARA*	12.4 (3.4)	11.5 (3)	17

* Genomic boundaries detected as peaks within regions of contiguous alteration are indented.

† False discovery-corrected p-value.

‡ RefSeq (hg17); in parentheses, human microRNAs.

§ Locus of alteration spanning known population CNV (see Methods), percent genomic coverage; in parentheses, mean sample count.

# Unknown: ambiguous direction of copy-number variant.

∥ Boundary spans multiple observed intragenic breakpoints.

& *TP53* is focally deleted (peak, chr17∶7501467–7574417), but high in analytical error on low-marker count segments.

$ Marginal evidence of germline alteration in only two normal samples.

ˆ Non-genic germline signal in six normal samples spanning only a fraction of the locus, terminating prior to genic content.

Broad and focal alterations stratified by event type. The genetic element of interest is selected from the total genic content of an alteration if it has previously observed somatic mutations in cancer (COSMIC), known oncogenes or tumor suppressors (CGP), implication in pathways altered in liposarcoma, or novel genes of interest for further study [43], [62], [63].

After *RB1* deletion, the next most common event was loss of 17p13.1 containing *TP53*. The pattern of p53 deletions includes both broad and focal hemizygous loss, and less common homozygous deletion. We also observe both deletion-associated and copy-neutral LOH at the p53 locus, consistent with previous reports (Figure S8). Given the potential therapeutic implications of the presence and type of p53 pathway alterations [41], we explored alternative lesions in the pathway in samples in which *TP53* is not deleted. *MDM2* (12q15) is gained in four such samples. Furthermore, upstream of *MDM2*, pLPS tumors seem to lack frequent alteration of *CDKN2A*, only two samples having evidence of deletion, only one of which is biallelic. However, neither *RB1* nor *TP53* are also deleted in these samples, confirming their functional redundancy. There are multiple other alterations targeting genes involved in these two well-studied pathways mediated by *RB1* and *p53*; G1/S phase transition during cell cycle progression, affecting *CCNE1*, *RB1*, *SKP2*, and *p53*, and DNA repair, including *FANCA*, *RAD1*, *RAD52*, *XRCC6*, and *MSH4*. A thorough discussion of each is outside the scope of this report.

In addition to these previously documented targets, RAE also identified frequent deletion of 17q11.2 containing *NF1* (neurofibromin 1). A total of nine pleomorphic liposarcomas have genomic loss at this locus, eight are hemizygous and one is homozygous. Germline deletions of *NF1* are frequently associated (∼50%) with another sarcoma, malignant peripheral nerve sheath tumors (MPNST), through its association with neurofibromatosis-1 [42]. The COSMIC database also lists mutations in *NF1* in multiple MPNSTs, colorectal carcinoma, and of course in neurofibromas, but none yet in liposarcoma [43].

RAE also identified a complex amplicon on 5p containing *CTNND2* (δ-catenin). Given the propensity of pleomorphic liposarcomas to metastasize, the amplification of δ-catenin is intriguing. δ-catenin functions as an adhesive junction-associated protein and its over-expression is associated with the down-regulation of E-cadherin in prostatic neoplasms [44]. Interestingly, E-cadherin (*CDH1*) is affected by frequent deletions of 16q22.1 (in 9 pLPS tumors; Table 1). E-cadherin is a metastasis suppressor gene mediating cell-cell adhesion. Its down-regulation is associated with an aggressive phenotype in multiple malignancies. Ostensibly, the amplification of δ-catenin may down-regulate E-cadherin in a deletion-independent manner. However, these are not mutually exclusive alterations, appearing together in multiple tumors. Deletion of E-cadherin is monoallelic in pLPS, suggesting the product of the remaining allele may be down-regulated by δ-catenin. However, E-cadherin inactivation can also occur through somatic mutation or promoter methylation [45]. Further work is needed to functionally validate the role of these alterations in pLPS.

A significant feature of liposarcomas is the deregulation of adipogenesis. RAE identified multiple alterations involving genes with known or putative roles in adipocyte differentiation. While *PPARγ* is the master transcriptional regulator of adipocyte differentiation, we noted only sporadic and sub-significant gains of the gene in pLPS. Nevertheless, there are multiple primary and secondary adipogenesis-related alterations. These include amplification of 19q spanning both *C/EBPα* and *C/EBPγ*. This amplification would contradict previously observed transcriptional down-regulation of *C/EBPα* in similar liposarcomas relative to normal fat. Due to the complexity and temporal features of adipocyte biology, it is either unlikely that genomic gains in these genes produces a dosage change, or they are partially regulated in an allele-dosage independent manner, such as with *JUN* amplification-mediated repression of *C/EBPβ* [46]. So we sought alteration in secondary, regulatory genes in adipogenesis. Indeed, there is an interesting deletion of 12p13.33 spanning several genes of interest. In all, 11 tumors had monoallelic deletion of this locus. We focus here on *WNT5B* (wingless-type MMTV integration site family, member 5B), which is implicated in the promotion of adipocyte differentiation through its inhibition of Wnt/β-catenin signaling [47]. In pre-adipocytes, the over-expression of *WNT5B* promotes adipogenesis through the reversal of Wnt signaling inhibition by *Wnt3a*-mediated nuclear translocation, and activation of β-catenin, described as an anti-adipogenic signal. Genomic deletion of *WNT5B* may imply tumor suppressor function, its loss relieving Wnt signaling of the competing inhibition, and allowing the pathway to exert its negative regulation of *PPARγ* and *C/EBPα* [48].

Another alteration potentially contributing to altered regulatory control of adipocyte differentiation is the monoallelic deletion of 22q13.1–q13.2 including *EP300* (EIA binding protein p300). This well-studied transcriptional co-activator is a putative tumor suppressor in multiple epithelial tumors and is part of a t(8;22)(p11;q13) translocation in acute myeloid leukemias (AML) [49]. *EP300* is necessary for the induction of *PPARγ* target genes, and its decrease suppresses *PPARγ* target gene expression [50]. This produces a concomitant down-regulation of preadipocyte differentiation. This implies, similar to *WNT5B*, tumor suppressor function in pleomorphic liposarcoma, though the status of the remaining allele is unknown. Considered together, these results indicate substantial alteration to pathways that exert either pro- or anti-adipogenic regulation in pleomorphic liposarcoma.

Finally, we wanted to design an approach that was suitable for exploring different classes of copy-number alteration. This includes alterations in pleomorphic liposarcomas that were also altered in a subset of high-quality matched normal DNA and subsequently classified as germline CNV (Figure 1). These are usually discarded *a priori*, as it remains unclear how best to resolve these as either benign variation, or variants conferring disease susceptibility. In pLPS, this included a focal amplification of 1p22.2 (from 91.33 to 91.46 mb, q-value = 0.021) spanning a single gene, *HFM1*, a putative human DNA helicase. Nine tumors were altered at the *HFM1* locus, as were two of their non-tumor DNA counterparts. Two additional matched normal samples were altered at the locus, but their respective tumors were not. Also, there is a dearth of population CNV at this locus. *hHFM1* encodes multiple conserved DNA/RNA helicase sequence motifs, is highly conserved with the *Saccharomyces cerevisiae* gene *Mer3*, a known helicase, and is preferentially expressed in germline tissues [51]. These results provide a hypothesis regarding a possible role of *HFM1* in susceptibility to soft-tissue sarcoma and extensive tumor aneuploidy.

A systematic integration of copy number changes with transcript array data and DNA sequence changes is currently underway in this and a larger set of sarcomas, which will provide a broader genetic landscape of adult soft-tissue sarcoma [Barretina J, Taylor BS et al. 2008, in preparation].

## Discussion

The present work details a novel method for mapping and assessing the significance of chromosomal abnormalities in cancer based on high-resolution array profiles of tens to many hundreds of tumor samples. As recurrent changes in a collection of tumors are more likely to represent candidate functional events than are those appearing to be randomly acquired, quantitative measures and statistical assessment are central to the method. For technical validation, we compared the performance of RAE to the GISTIC method in a large collection of solid tumors including lung adenocarcinomas and primary and secondary gliomas. As a discovery application, we used RAE to determine the spectrum of copy-number alteration in pleomorphic liposarcoma patients. Separately, we have used this method to identify significant alterations in additional glioblastomas, thyroid carcinomas, localized and metastatic prostate cancers, and additional subtypes of soft-tissue sarcoma (unpublished work). In comparison with other methods, the key advantages of our approach are that it: (i) treats the analysis of genomic gain and loss differently, distinguishing between four classes of alteration in a manner that reflects their biological differences, (ii) each of these four scoring models are sample-specific, adapted to individual tumors to account for their differences, (iii) we use soft discrimination in lieu of hard thresholds for improved signal extraction, and (iv) in a collection of tumors, it generates a random aberration model using a background of more realistic segmental DNA, rather than unrealistically independent array markers. The RAE method is flexible; it is suitable for analyzing data from any array platform or cancer type varying from genetically simple to chaotic. This includes the accommodation of new chip types or increased resolution. Also, RAE is modular. For example, after the generation of the UBP, alternative algorithms can be substituted to achieve varying analytical goals. Or, while modifying the initial phases of the framework, the later components are valid and applicable for the analysis of copy number changes produced by next-generation sequencing.

Of course, the distinction between *driver* events and biological neutral *passenger* changes is difficult to achieve definitively. While a clonal population of tumor cells selects for changes conferring a growth advantage (driver), it also propagates non-functional (neutral) alterations. Indeed, recent work in a related domain, large-scale sequencing of solid tumors, demonstrates the statistical challenges in attempting to make this distinction [52], [53], [54], [55], [56], [57]. For DNA copy number, these challenges result from the complexity and tumor-to-tumor variability of the data, even in clinically well-defined tumor types. For realistic statistical assessment in a collection of tumors, we feel that an individual-tumor noise model producing sample-specific effective thresholds is more appropriate than a single global threshold and that separate treatment of different types of copy number changes is very useful in practice. We are also confident that random perturbations using the unified breakpoint profile (UBP) provide a much more realistic background (null) model than random perturbations of a much larger number of (linked) individual probe data points. However, the count of truly independent regions of change is likely still lower than implied by the UBP model. Therefore, as background models become more sophisticated in their modeling of random aberrations, one should explicitly include additional mechanisms potentially mediating copy number change (prior to any selection). This includes perhaps a proxy of non-homologous end joining (NHEJ), which is a prominent feature of rearrangements in tumor cells, as well as other mechanisms. Finally, no gold standard of experimentally validated alterations in a large tumor collection exists, which is a necessary step to elucidate the features of varying methods and the positive and negative results they generate.

DNA copy number data has clear limitations. Alterations in most cancers are large, spanning many tens if not hundreds of genes, many of which are likely not involved in oncogenesis. Identifying the small number of targets from these events even across many tumors is difficult. Additionally, oncogenic activation by mechanisms other than amplification or deletion, like mutation or epigenetic silencing is also important. Therefore, major advances in our understanding of cancer genetics will likely come from integrating copy number data with additional genomic data types and, of course, functional genetic experiments, both large-scale and hypothesis driven.

## Materials and Methods

### Array pre-processing, segmentation, and quality control

Genomic DNA was extracted from tumor and either normal adjacent fat, muscle, or blood and genotyped with Affymetrix 250 K oligonucleotide arrays (StyI) according to manufacturers' specifications. Raw data from the 270 HapMap individuals hybridized to the Affymetrix GeneChip Mapping 500 K array set were downloaded from NCBI GEO (accession number GSE5173) [9]. From the 210 unrelated individuals of the latter, we randomly chose a set of 140 individuals as the reference normal dataset (CEU: 20 Utah residents with northern and western European ancestry; CHB: 45 Han Chinese in Beijing; JPT: 45 Japanese in Tokyo; YRI: 30 Yoruba in Ibadan, Nigeria; Methods S1, Table S1, Figure S2). This set was randomly partitioned into two subsets (HapMap.A: n = 40; HapMap.B: n = 100). All Affymetrix data was processed from original CEL files with the Affymetrix Chromosomal Copy Number Analysis Tool (CNAT 4.0, 1.5.6_v3.1). Genotyping, probe-level signal intensity normalization (quantile), and copy-number quantification of tumor, matched normal, and HapMap.B samples were generated from the unpaired sample workflow (CNAT) with HapMap.A samples serving as the internal control, and with default parameters and a Gaussian smoothing bandwidth of zero (Methods S1). Copy number was converted to a tumor-to-normal log_2_ ratio by subtracting from the tumor signal at each marker the median signal intensity of HapMap.B samples. Samples were then segmented with the Circular Binary Segmentation algorithm (CBS; α = 0.01, permutations = 10,000, undo.splits = none) [58]. We normalized segmentation output in the following way. First, we calculated a measure of sample-specific probe-level noise equal to the difference in signal between adjacent markers (probes) on the array: for each sample *j*, the derivative noise *DN_j_* parameter is
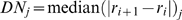
(1)where *r_i_* is the log_2_ ratio of signal for the *i^th^* marker. We used the median because of its robustness to non-noise outliers that correspond to valid segmentation breakpoints. The density distribution of segmentation (segment means mapped to their resident probes and therefore scaled by size) was calculated with a width based on the value of *DN_j_*, and mode-centered per sample. The technical failure criterion was a tumor genotype call rate (when available, Affymetrix-only) of less than 85%.

### The soft discriminator model

Let *a_ij_* represent normalized log_2_ copy number at each marker *i* in sample *j*; we derived four sigmoid detectors (*S*), one for each alteration state (component) *k* where *k* = {A_0_, A_1_, D_0_, D_1_} and correspond to single-copy gain, amplification, hemizygous loss, and homozygous deletion respectively, such that:
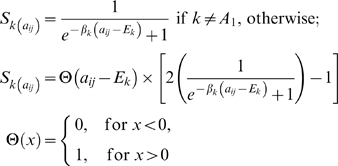
(2)


Similar to the logistic function, *S_k_*(·) maps log_2_ ratios to values bounded by 0 and 1 subject to parameters *E_k_* and *β_k_*. We derived four sets of these parameters, one for each alteration state *k*, which were adaptively determined per sample (Methods S1). The truncated sigmoid of A_1_ (Eq. 2) was designed to integrate *a_ij_*, assigning a value that was monotonic to the original magnitude of the event.

To transition from scoring markers to scoring regions, and for each region and in each sample, we calculated the weighted arithmetic mean for each *S_k_* across the *m* markers in that region. We then calculated the mean *S_k_* across all samples in each region of the UBP. These individual *S_k_* scores were also used to compute summary scores of total gain and loss, for which we used the L_2_ norm:
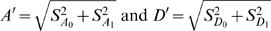
(3)


This scoring approach is flexible. Any of the components (*k*) can be statistically assessed independently, combined as above (used in this paper), or weighted with coefficients (data not shown) such that one *S_k_* is perceived as substantively more important for the desired event detection than another.

### Unified Breakpoint Profile (UBP)

To generate a common division of the genome for between-tumor analysis, we combined segmentation breakpoints from all tumors. For segments of ≥3 markers, we extracted the unique set of start positions. To each of these we assigned a terminus position that is the marker directly 5′ of the next adjacent start site. This simple procedure covers all segments and samples, is a common division of each chromosome derived directly from original segmentation breakpoints, and is not subject to an artificial length scale. This latter point avoids unnecessary and likely inaccurate assumptions about array resolution or alteration size that are study/tumor-type-specific. We note some variability exists in the position of similar breakpoints between tumors. While repetitive breakpoints between tumors were inherited as a single unique position, we frequently see breakpoints with similar but not identical genomic positions. From tumor to tumor, these vary by a single marker in either the 5′ or 3′ direction. This is likely caused by the finite resolution of the experiment such that the true breakpoint is proximal to the markers on the array. Operationally, this produces either (i) an isolated single-marker event during between-tumor aggregation, or (ii) a contiguous stretch of single-marker events. We therefore merged the former to the physically adjacent 3′ region, while the latter were consolidated as an independent region.

Finally, problematic segmentation will result in a sub-standard UBP. Two problematic sample types exist. Non-tumor cell contamination tends to cause under-segmentation, producing large segments of reduced mean amplitude and fewer disease-associated breakpoints. Conversely, excessive array noise can produce over- or hyper-segmentation (Methods S1, Figure S6). To account for both, we derived the distribution of segment counts for a cohort and outlier samples (with too few or too many segments) were excluded from UBP generation. From our recent experience with an internal repository of 1,426 tumors spanning 11 tumor types of mixed karyotypic and genetic complexity, a median of ∼4% of tumor samples in any individual study were excluded by this step (unpublished work). Nevertheless, after UBP generation, these samples were added back and assessed because even poorly segmented samples may bear an underlying disease-specific profile (Figure S7).

### Null model generation

We used human recombination hotspots (n = 32,996) predicted from phase II HapMap data (release 21) [27], [59] to further divide the genome prior to generating the null (background) model. However, there exists systematic and fine-scale heritable variation in hotspot use [60]. Therefore, to arrive at a generally valid background model, we randomized the positions of these hotspots in a manner sensitive to their physical distribution in the genome. Let *R(h)* represent the distribution of hotspots on a non-acrocentric chromosome arm with a width equal to the median distance between all hotspots on that arm (ranging from 38 to 63 kb across the 39 arms), with *R(h)* normalized to range between 0 and 1. We randomly selected a genomic position *x_p_* on the arm and a value of *u_p_* from the uniform distribution U[0,1]. If *u_p_*<*R(h_p_)*, then *x_p_* was accepted, otherwise, it was rejected and the process was repeated. This continued until the count of accepted positions, i.e., random genomic positions according to *R(h)*, equaled the real count of hotspots on that chromosome arm. This process preserved, on average, the spatially non-uniform structural features of the original distribution (Figure 3B) [26], [27].

To generate a usable background breakpoint pattern, these randomized hotspots were mapped to the marker nearest each in the genome, which was then used as the position of random cleavage. To prevent excessive fracturing of the original tumor segmentation profile, we added a breakpoint at the hotspot site only for segments of size greater than the median size of all segments by disease type. Furthermore, a breakpoint was added only when the resulting segments were ≥2 markers in length and had a size of no less than one half the original global median segment size. Each split inherited the copy number assigned to its parental segment, which maintains the correlation in copy number. This procedure effectively maintained the mode of the distribution of event sizes, allowing for a permutation of copy number values and not positions, without decoupling a possible relationship between amplitude and size (data not shown) (Figure 3C). This is desirable as a measure of model simplicity, as permuting variable-sized fragments of DNA between differently sized chromosomes is complex. From this cleaved profile of tumor segmentation, we generated a new unified breakpoint profile and scored each new region as before. These steps were repeated for every 1,000 permutations of the null model. Each of these permutations was a genome-wide randomization in each tumor of the regenerated UBP and the scores assigned to these regions by each of the four components.

### Regions of Interest (ROI)

As discussed in the text, the first stage of the ROI algorithm identifies all contiguous regions of statistically significant alteration. We describe the second stage of the algorithm here. First, in each of these larger regions spanning more than one region of the UBP, we detect peaks in only the subset of regions exceeding an order-of-magnitude greater significance (q≤0.001). This prevents over-sensitivity of peak detection, i.e., regions of marginally higher significance (0.001<q<0.01) were deemed neither likely to be differentially selected for by oncogenesis nor to reflect a substantial increase in either recurrence or magnitude. Second, for eligible regions (q≤0.001), we detected peaks in the summary score (Eq. 3) and not in their p- or q-values. This is because the number of random samples one can practically generate with available computer resources limits the smallest p-value. This would restrict our ability to identify tumor events with scores greater than the maximum scoring random aberration of the background model (assigning a p-value corresponding to 1/(*N_p_*+1) to the tumor event, where *N_p_* is the count of random aberrations) (dashed curve in Figure 4). Because this affects regions of greatest interest, we determined peaks with a simple detector of local maxima in A′ and D′ as these are monotonic with the p-value and maximally resolved for any region of the UBP. These peaks, especially in analyses of uncommon tumor types, are sensitive to the level of error in the system (see text). Therefore we analytically derive a unique and symmetric value of error for each region of the UBP and this determined the sensitivity of peak detection in two ways (Methods S1). First, the detector identifies zero, one, or more peaks (identified with red plus in Figure 4) based on a shoulder sensitivity parameter that we set to two times the median analytical error of all UBP regions in the larger event (all error bars above the peak threshold in Figure 4). Second, we assumed that two or more statistically significant and physically adjacent regions that are assigned summary scores that lie within the error of the other likely do not define unique and independent events. Therefore, regions adjacent to a peak are merged with that locus if their summary score fell within the error bar of the peak (regions with red error bars in Figure 4).

### Analytical error in the multi-component model

Error was calculated for each of the four components *k* and for the final summary scores (A′, D′). It was computed from the standard deviations of the original probe-level data in each segment from each sample spanning a given region of the unified breakpoint profile (UBP) (details in Methods S1).

### Germline variation

Patient-matched normal samples were processed identically to the tumors. We plotted the distribution of normalized segmentation and excluded normal samples from analysis if they had; (i) gross asymmetry, as either negative or positive skew in the diploid peak producing high proportions of non-neutral copy number, or (ii) samples with incoherently multimodal distributions of segmentation. We parameterized and calculated A_0_ and D_0_ for the remaining normal samples. We then identified regions of apparent CNV (polymorphisms in the human population) as loci with A_0_ or D_0_ ≥0.50 in two or more samples. These were removed from genome-wide significance plotting, and their genomic coverage spanning statistically significant tumor regions was calculated and reported (Tables S2–S3).

### Human structural variation

Loci of structural and copy-number variation were obtained from the Database of Genomic Variants (DGV) at The Center for Applied Genomics (TCAG; http://projects.tcag.ca/variation) [61]. Autosomal copy-number variants profiled in any of 35 studies of human genomic variation (version 3) were included in the screen.

### Genome Mapping

All genomic coordinates were standardized to NCBI build 35 (University of California, Santa Cruz (UCSC) May 2004 (hg17) assembly) of the human genome.

### Availability

To support the analysis of copy number data, RAE is freely available to the research community, located at: http://cbio.mskcc.org/downloads/rae

## Supporting Information

Methods S1 Supplementary methods, results, notes, and references
(0.12 MB DOC)

Table S1 Individuals of the HapMap collection included in the reference normal
(0.31 MB DOC)

Table S2 Additional genomic gain/amplification in pleomorphic liposarcoma
(0.10 MB DOC)

Table S3 Additional genomic loss/deletion in pleomorphic liposarcoma
(0.13 MB DOC)

Table S4 Comparison of significant events in the lung and glioma datasets.
(0.05 MB DOC)

Table S5 Values of Ek for lung and glioma datasets
(0.04 MB DOC)

Figure S1 Diploid copy number between patient-matched non-tumor DNA and HapMap reference normal. The distribution of segment means weighted by their size for a randomly chosen HapMap individual from the copy-called partition (HapMap.B) of the reference normal (blue) and three tumor-matched normal samples (gray). The tight and symmetric diploid peak of the HapMap individual is juxtaposed to poorly behaved distributions of patient-matched normal DNA copy-number data.
(0.33 MB TIF)

Figure S2 Confirming population genetic structure of HapMap reference normal. Population clustering of n = 140, 40, and 100 HapMap individuals from the partitions of the reference normal, assuming three ancestral populations (k = 3; triangle plots). Clustering is based on the 67 non-redundant biallelic CNVs from Redon et al. [ref. S3] and repeated on only those 31 genotypes derived from the Affymetrix early access platform (as indicated).
(0.85 MB TIF)

Figure S3 Detecting an intermediate phenotype in copy number. In a single tumor, monosomic chromosome 15 is adjacent to intermediate signal on chromosome 14 (probe-level data in gray, segmentation in blue). At right is the density distribution of autosomal segmentation weighted by event size (same annotation as in Figure S5). The peak representing the medial loss signal (green plus) may arise from either multiple tumor-cell populations in the DNA isolate, or allele-specific copy number (dark red sigmoid; D_0_ parameterized with indicated values of *E* and *β*).
(0.62 MB TIF)

Figure S4 Relationship between probe-level and segmentation noise. The two very different types of noise associated with array-based copy number data. We can view the segmentation algorithm as a de-noising step that attempts to remove noise in probe-level measurements to accurately estimate the local copy number. Here, we measure the efficiency of this noise reduction by calculating the change in entropy from probe-level data to the entropy for segmentation values (means). To precisely define the entropy, we first compute the probability density histograms of both the probe-level and segmentation data using the R density function with a fixed bandwidth, limits (from −1 to 1 in log2 ratio units), and a fixed number of bins (2048). The entropy is then defined as shannon entropy where *pi* is the probability for each of the 2048 bins. When segmentation works properly, we observe a large change in entropy [where Δ*S* = *S*(*r*)−*S*(*m*)] from the probe-level data (*r*) to the segmentation values (*m*). However, the phenomenon of hyper-segmentation (which we have observed in many tumors; Methods S1, Figure S6) occurs when the segmentation algorithm generates a large number segments (far larger a number than are likely to be in the real data). As one would expect there is a simple relationship between this over-segmentation and the reduction in the change in entropy. However, these poorly behaved tumors, as measured by over-segmentation, seem to be uncorrelated with the low-level noise in their probe-level data as measured by the derivative noise (*DN*; see Methods). The derivative noise is represented by the size of the circles, where the radius in proportional to the *DN*. Notice that *DN* is roughly uniformly scattered along the Δ*S* x-axis.
(0.72 MB TIF)

Figure S5 Diversity and heterogeneity of tumor profile. At left is probe-level (gray) and segmentation data (blue) for 11 chromosomes (indicated, centromere in red). At right is the density distribution of segmentation means weighted by their size (22 autosomes). For each, the sigmoid detecting single-copy gain (A_0_) and hemizygous loss (D_0_) are indicated (red and blue respectively, parameters labeled). The diploid peak is identified at zero in log_2_ copy-number (gray line) and the half-maximum values of the diploid peak are identified (dotted gray lines). (a) Tumor with a symmetric and well-behaved diploid peak as well as smaller peaks of detectable signal (at left, on chromosomes 8p, 9, 10, 11, and 15). (b) A hyper-segmented tumor with asymmetry in the diploid peak exclusively in deletion, challenging the choice of *E* for single-copy loss (D_0_). Nested signal can be detected in the form of whole-chromosome loss of chr15, an event detected in panel A as well. (c) A tumor having a highly complex and reduced-quality segmentation profile, gross asymmetry in both gain and loss, an ill-defined diploid peak, and lacks discernible features for the selection of parameters for its transformation in either gain or loss.
(1.69 MB TIF)

Figure S6 Hyper-fragmentation of copy number segmentation. (a) Normalized probe-level (gray) and segmentation (green) of chromosome 1 in a single tumor displaying a hyper-fragmentation pattern. (b) The same probe-level signal as in panel A, superimposed with a spatially averaged (bandwidth of ∼601 kb) version. Convolved trace indicates a non-disease related periodicity in signal likely the source of hyper-segmentation (Methods S1).
(0.94 MB TIF)

Figure S7 Varying segmentation quality between tumors does not preclude detection. Here, three chromosomes (14, 15, and 16) in two tumors, probe-level (orange and light blue) and segmentation data (red and dark blue, respectively). A high-quality segmentation result (dark blue) identifies monoallelic loss of the q-arm of chromosome 15. The lower-quality hyper-segmentation (red) also includes the 15q loss (highlighted). This motivates the conditional inclusion of both samples during scoring and assessment, but not the latter during UBP derivation.
(0.93 MB TIF)

Figure S8 Genomic deletion and observed loss-of-heterozygosity for the *p53* locus. Independent hierarchical clustering of copy number (segmentation, left) and LOH (paired, right) for 2.2 mb of 17p13.1 (columns are samples, rows are markers) indicates two patterns of alteration in pleomorphic liposarcomas. Deletion-associated LOH for p53 in three tumors with either broad or focal deletion (lines connect corresponding samples; black), and copy-neutral LOH (green).
(2.93 MB TIF)

Figure S9 The affect of normalization on segmentation profiles. Here, the median of original segmentation of each glioma tumor (x-axis) and the distance (offset) of the mode of the diploid peak from log_2_ = 0 are plotted. While the median is a reasonable approximation for the diploid feature of most tumors, in a subset of tumors, the mode of the diploid peak and the median of un-normalized segmentation are substantially different.
(0.26 MB TIF)

Figure S10 Difference between symmetric global threshold and *Ek* from the individual tumor noise model. Here, the *Ek* value for A_0_ and D_0_ (single-copy gains and losses respectively) are shown for all 141 tumors of the glioma dataset. This indicates that in the majority of tumors, the detector for single-copy events in RAE is more stringent than was the original log_2_ global threshold used by the original study (red dotted lines). This is responsible for the global reduction in alteration frequencies in the RAE analysis.
(0.26 MB TIF)

## References

[1] Albertson DG, Collins C, McCormick F, Gray JW (2003). Chromosome aberrations in solid tumors.. Nat Genet.

[2] Druker BJ, Talpaz M, Resta DJ, Peng B, Buchdunger E (2001). Efficacy and safety of a specific inhibitor of the BCR-ABL tyrosine kinase in chronic myeloid leukemia.. N Engl J Med.

[3] Engelman JA, Zejnullahu K, Mitsudomi T, Song Y, Hyland C (2007). MET amplification leads to gefitinib resistance in lung cancer by activating ERBB3 signaling.. Science.

[4] Lynch TJ, Bell DW, Sordella R, Gurubhagavatula S, Okimoto RA (2004). Activating mutations in the epidermal growth factor receptor underlying responsiveness of non-small-cell lung cancer to gefitinib.. N Engl J Med.

[5] Paez JG, Janne PA, Lee JC, Tracy S, Greulich H (2004). EGFR mutations in lung cancer: correlation with clinical response to gefitinib therapy.. Science.

[6] Pao W, Miller V, Zakowski M, Doherty J, Politi K (2004). EGF receptor gene mutations are common in lung cancers from “never smokers” and are associated with sensitivity of tumors to gefitinib and erlotinib.. Proc Natl Acad Sci U S A.

[7] Slamon DJ, Clark GM, Wong SG, Levin WJ, Ullrich A (1987). Human breast cancer: correlation of relapse and survival with amplification of the HER-2/neu oncogene.. Science.

[8] Pinkel D, Albertson DG (2005). Array comparative genomic hybridization and its applications in cancer.. Nat Genet.

[9] Redon R, Ishikawa S, Fitch KR, Feuk L, Perry GH (2006). Global variation in copy number in the human genome.. Nature.

[10] Emanuel BS, Saitta SC (2007). From microscopes to microarrays: dissecting recurrent chromosomal rearrangements.. Nat Rev Genet.

[11] Lai WR, Johnson MD, Kucherlapati R, Park PJ (2005). Comparative analysis of algorithms for identifying amplifications and deletions in array CGH data.. Bioinformatics.

[12] Willenbrock H, Fridlyand J (2005). A comparison study: applying segmentation to array CGH data for downstream analyses.. Bioinformatics.

[13] Aguirre AJ, Brennan C, Bailey G, Sinha R, Feng B (2004). High-resolution characterization of the pancreatic adenocarcinoma genome.. Proc Natl Acad Sci U S A.

[14] Tonon G, Wong KK, Maulik G, Brennan C, Feng B (2005). High-resolution genomic profiles of human lung cancer.. Proc Natl Acad Sci U S A.

[15] Diskin SJ, Eck T, Greshock J, Mosse YP, Naylor T (2006). STAC: A method for testing the significance of DNA copy number aberrations across multiple array-CGH experiments.. Genome Res.

[16] Guttman M, Mies C, Dudycz-Sulicz K, Diskin SJ, Baldwin DA (2007). Assessing the significance of conserved genomic aberrations using high resolution genomic microarrays.. PLoS Genet.

[17] Shah SP, Lam WL, Ng RT, Murphy KP (2007). Modeling recurrent DNA copy number alterations in array CGH data.. Bioinformatics.

[18] Beroukhim R, Getz G, Nghiemphu L, Barretina J, Hsueh T (2007). Assessing the significance of chromosomal aberrations in cancer: Methodology and application to glioma.. Proc Natl Acad Sci U S A.

[19] National Cancer Institute NHGRI (2007). The Cancer Genome Atlas.

[20] Hahn SA, Seymour AB, Hoque AT, Schutte M, da Costa LT (1995). Allelotype of pancreatic adenocarcinoma using xenograft enrichment.. Cancer Res.

[21] Lindblad-Toh K, Tanenbaum DM, Daly MJ, Winchester E, Lui WO (2000). Loss-of-heterozygosity analysis of small-cell lung carcinomas using single-nucleotide polymorphism arrays.. Nat Biotechnol.

[22] Freeman JL, Perry GH, Feuk L, Redon R, McCarroll SA (2006). Copy number variation: new insights in genome diversity.. Genome Res.

[23] Inoue K, Lupski JR (2002). Molecular mechanisms for genomic disorders.. Annu Rev Genomics Hum Genet.

[24] Lupski JR (2004). Hotspots of homologous recombination in the human genome: not all homologous sequences are equal.. Genome Biol.

[25] Sebat J, Lakshmi B, Troge J, Alexander J, Young J (2004). Large-scale copy number polymorphism in the human genome.. Science.

[26] Myers S, Bottolo L, Freeman C, McVean G, Donnelly P (2005). A fine-scale map of recombination rates and hotspots across the human genome.. Science.

[27] Frazer KAPI, Ballinger DG, Cox DR, Hinds DA, Stuve LL (2007). A second generation human haplotype map of over 3.1 million SNPs.. Nature.

[28] Benjamini Y, Hochberg Y (1995). Controlling the False Discovery Rate: A Practical and Powerful Approach to Multiple Testing.. Journal of the Royal Statistical Society Series B (Methodological).

[29] Carrasco DR, Tonon G, Huang Y, Zhang Y, Sinha R (2006). High-resolution genomic profiles define distinct clinico-pathogenetic subgroups of multiple myeloma patients.. Cancer Cell.

[30] Perry GH, Ben-Dor A, Tsalenko A, Sampas N, Rodriguez-Revenga L (2008). The fine-scale and complex architecture of human copy-number variation.. Am J Hum Genet.

[31] Weir BA, Woo MS, Getz G, Perner S, Ding L (2007). Characterizing the cancer genome in lung adenocarcinoma.. Nature.

[32] Mack TM (1995). Sarcomas and other malignancies of soft tissue, retroperitoneum, peritoneum, pleura, heart, mediastinum, and spleen.. Cancer.

[33] Fletcher CDM, Unni KK, Mertens F, World Health Organization, International Academy of Pathology (2002). Pathology and genetics of tumours of soft tissue and bone.

[34] Ladanyi M, Antonescu CR, Dal Cin P, Weiss SW, Goldblum JR (2008). Cytogenetic and Molecular Genetic Pathology of Soft Tissue Tumors.. Enzinger & Weiss's Soft Tissue Tumors.

[35] Fritz B, Schubert F, Wrobel G, Schwaenen C, Wessendorf S (2002). Microarray-based copy number and expression profiling in dedifferentiated and pleomorphic liposarcoma.. Cancer Res.

[36] Rieker RJ, Joos S, Bartsch C, Willeke F, Schwarzbach M (2002). Distinct chromosomal imbalances in pleomorphic and in high-grade dedifferentiated liposarcomas.. Int J Cancer.

[37] Dalal KM, Kattan MW, Antonescu CR, Brennan MF, Singer S (2006). Subtype specific prognostic nomogram for patients with primary liposarcoma of the retroperitoneum, extremity, or trunk.. Ann Surg.

[38] Singer S, Socci ND, Ambrosini G, Sambol E, Decarolis P (2007). Gene expression profiling of liposarcoma identifies distinct biological types/subtypes and potential therapeutic targets in well-differentiated and dedifferentiated liposarcoma.. Cancer Res.

[39] Helman LJ, Meltzer P (2003). Mechanisms of sarcoma development.. Nat Rev Cancer.

[40] Wong FL, Boice JD, Abramson DH, Tarone RE, Kleinerman RA (1997). Cancer incidence after retinoblastoma. Radiation dose and sarcoma risk.. Jama.

[41] Ambrosini G, Sambol EB, Carvajal D, Vassilev LT, Singer S (2007). Mouse double minute antagonist Nutlin-3a enhances chemotherapy-induced apoptosis in cancer cells with mutant p53 by activating E2F1.. Oncogene.

[42] King AA, Debaun MR, Riccardi VM, Gutmann DH (2000). Malignant peripheral nerve sheath tumors in neurofibromatosis 1.. Am J Med Genet.

[43] Bamford S, Dawson E, Forbes S, Clements J, Pettett R (2004). The COSMIC (Catalogue of Somatic Mutations in Cancer) database and website.. Br J Cancer.

[44] Lu Q, Dobbs LJ, Gregory CW, Lanford GW, Revelo MP (2005). Increased expression of delta-catenin/neural plakophilin-related armadillo protein is associated with the down-regulation and redistribution of E-cadherin and p120ctn in human prostate cancer.. Hum Pathol.

[45] Strathdee G (2002). Epigenetic versus genetic alterations in the inactivation of E-cadherin.. Semin Cancer Biol.

[46] Mariani O, Brennetot C, Coindre JM, Gruel N, Ganem C (2007). JUN Oncogene Amplification and Overexpression Block Adipocytic Differentiation in Highly Aggressive Sarcomas.. Cancer Cell.

[47] Kanazawa A, Tsukada S, Kamiyama M, Yanagimoto T, Nakajima M (2005). Wnt5b partially inhibits canonical Wnt/beta-catenin signaling pathway and promotes adipogenesis in 3T3–L1 preadipocytes.. Biochem Biophys Res Commun.

[48] Rosen ED, MacDougald OA (2006). Adipocyte differentiation from the inside out.. Nat Rev Mol Cell Biol.

[49] Ida K, Kitabayashi I, Taki T, Taniwaki M, Noro K (1997). Adenoviral E1A-associated protein p300 is involved in acute myeloid leukemia with t(11;22)(q23;q13).. Blood.

[50] Takahashi N, Kawada T, Yamamoto T, Goto T, Taimatsu A (2002). Overexpression and ribozyme-mediated targeting of transcriptional coactivators CREB-binding protein and p300 revealed their indispensable roles in adipocyte differentiation through the regulation of peroxisome proliferator-activated receptor gamma.. J Biol Chem.

[51] Tanaka K, Miyamoto N, Shouguchi-Miyata J, Ikeda JE (2006). HFM1, the human homologue of yeast Mer3, encodes a putative DNA helicase expressed specifically in germ-line cells.. DNA Seq.

[52] Wood LD, Parsons DW, Jones S, Lin J, Sjoblom T (2007). The Genomic Landscapes of Human Breast and Colorectal Cancers.. Science.

[53] Sjoblom T, Jones S, Wood LD, Parsons DW, Lin J (2006). The consensus coding sequences of human breast and colorectal cancers.. Science.

[54] Rubin AF, Green P (2007). Comment on “The consensus coding sequences of human breast and colorectal cancers”.. Science.

[55] Greenman C, Stephens P, Smith R, Dalgliesh GL, Hunter C (2007). Patterns of somatic mutation in human cancer genomes.. Nature.

[56] Getz G, Hofling H, Mesirov JP, Golub TR, Meyerson M (2007). Comment on “The consensus coding sequences of human breast and colorectal cancers”.. Science.

[57] Forrest WF, Cavet G (2007). Comment on “The consensus coding sequences of human breast and colorectal cancers”.. Science.

[58] Olshen AB, Venkatraman ES, Lucito R, Wigler M (2004). Circular binary segmentation for the analysis of array-based DNA copy number data.. Biostatistics.

[59] McVean GA, Myers SR, Hunt S, Deloukas P, Bentley DR (2004). The fine-scale structure of recombination rate variation in the human genome.. Science.

[60] Coop G, Wen X, Ober C, Pritchard JK, Przeworski M (2008). High-Resolution Mapping of Crossovers Reveals Extensive Variation in Fine-Scale Recombination Patterns Among Humans.. Science.

[61] Iafrate AJ, Feuk L, Rivera MN, Listewnik ML, Donahoe PK (2004). Detection of large-scale variation in the human genome.. Nat Genet.

[62] Futreal PA, Coin L, Marshall M, Down T, Hubbard T (2004). A census of human cancer genes.. Nat Rev Cancer.

[63] Major JE (2007). Genomic mutation consequence calculator.. Bioinformatics.

