# Supplementary material for: Functional Copy-Number Alterations in Cancer
Source: PLoS One. 2008 Sep 11;3(9):e3179. doi: 10.1371/journal.pone.0003179 (PMC2527508; doi:10.1371/journal.pone.0003179)
Supplement: Methods S1 — Supplementary methods, results, notes, and references (0.12 MB DOC) [file pone.0003179.s001.doc]

Supplementary Text For

**Functional Copy-number Alterations in Cancer**

Barry S. Taylor, Jordi Barretina, Nicholas D. Socci, Penelope DeCarolis, Marc Ladanyi, Matthew Meyerson, Samuel Singer, Chris Sander

**Contents**

1. **Supplementary Methods**
   1. The HapMap reference normal
   2. Parameterization of E_k_ and β_k_ in the multi-component model
   3. Analytical derivation of error
2. **Supplementary Results**
3. Tumor Sequencing Project, Lung Adenocarcinoma
4. Glioma
5. **Supplementary Notes**
6. Operational benefit of the unified breakpoint profile (UBP)
7. Hyper-fragmentation during segmentation
8. Consequences of alternative permutation models
9. **Supplementary References**

**I. Supplementary Methods**

**A. The HapMap reference normal**

The 270 samples in the HapMap collection are heretofore-healthy individuals, rigorously selected and systematically analyzed in multiple data modalities. Nevertheless, the explicit familial structure in two of the four sub-populations violates the assumption of independence across samples in our analytical context. So, a subset of the cohort (n=140) was selected to enforce independence between individuals. All samples of the unrelated Japanese and Chinese populations (n=90) were selected along with a randomly chosen family member from the parent/offspring trios of Yoruba and CEPH populations (n=50, Table S1).

To confirm this randomized selection did not introduce bias in the population genetic structure of the HapMap collection, which might then compromise the reference normal, we performed population clustering similar to that of Redon et al. [1,2,3]. The 67 non-redundant biallelic copy-number variant (CNV) genotypes from the former were used to cluster three partitions of the HapMap collection (reference, n=140; HapMap.A, n=40; HapMap.B, n=100). Discrimination between ancestral populations (k=3) was obtained in each of the three subsets of individuals with this marker set (Figure S2). We repeated the clustering with a reduced marker set, those 31 biallelic CNV genotypes culled exclusively from the 500K EA platform, the platform data used in our reference set. This reduced the accuracy of ancestral assignment. This is expected as previous studies have demonstrated a drop in ancestral assignment with reduced power and small marker sets [1]. Nevertheless, the optimal clustering of ancestries relative to previously published results on a larger fraction of the same collection gave us confidence in an unbiased selection of our reference set, which we then partition into two subsets. Partition sizes for HapMap.A and HapMap.B were chosen to exceed recommended requirements for quantification and normalization algorithms. For the analysis of Agilent aCGH data, this step is skipped.

**B. Parameterization of *E_k_* and *β_k_* in the multi-component model**

For each tumor, two independent parameters are needed for each of the four detectors (*S*), one for each alteration status (component) *k* where *k* = {A_0_, A_1_, D_0_, and D_1_}: a location parameter (*E_k_*) and the slope (*β_k_*). This is a total of eight parameters per sample. In the ideal case, the parameters for single-copy gain (A_0_) and hemizygous loss (D_0_) are chosen such that the location falls between the peak of diploid copy number and the corresponding peaks of signal for each. Specifically, we derive frequency distribution plots for the log_2_ segmentation means (examples; Figures S3 and S5). While there are several ways to plot this density distribution, we used the following procedure. Each probe is assigned a mean and standard deviation value from the segment to which it belongs. If *m_i_* is the segment mean and *s_i_* is the standard deviation for each probe *i*, we then sample for each probe from a Gaussian distribution with mean *m_i_* and standard deviation *s_i_* to calculate *r_i_*, which has the segment mean value, but with noise added back to represent the intrinsic noise of the segmentation (not the probe-level noise). We then use a standard density function (R 2.6.1) to plot the frequency distribution of *r_i_* from a log_2_ ratio value of -1 to 1 with *n* (the number of density points) equal to 2,048, and a bandwidth adjustment equal to 2. For well-behaved tumors, the largest peak should correspond to a log_2_ ratio of zero (diploid) with two well-defined peaks adjacent to the central peak corresponding to the gain and loss of one copy respectively. There is no unique definition of a well-defined diploid peak, but we first try to detect the largest peak that falls between 2× and 0.5× the derivative noise (*DN*; see text, Eq. 1) for a tumor and insist the peak has a magnitude greater than the half-maximum point. The half-maximum point is one way to measure the noise level of the segmentation result and is the log R-value at which the density is one-half the value of the diploid peak. Note the half-maximum is often asymmetric such that the value on the deletion side may be different (and often larger) than the value on the amplification side. If there is a peak that meets these criteria (if there are several, we choose the largest in magnitude), then if *X_p_* is the log_2_ value of the peak and *H_d_* is the log R-value for the half-maximum point for deletion, then *E* for D_0_ is the average of these two values and β is chosen such that the value of the sigmoid function is equal to 10^-3^ at *H_d_* (where *S*(*H_d_*; E_0_, β)=0.001). The same is done for A_0_ using the corresponding values for gain.

In the event that no such peak meets the above criteria, then we use the following to pick the value of *E*. We fix *β* at a constant value of 20 and find the value of *E* that again sets the sigmoid value to 10^-3^ at *H_d_* or *H_a_* (where *S*(*H_d_*; *E*, 20)=0.001). The motivation here is to recover some signal, but to be aggressive in suppressing noise from the problematic segmentation of the data.

In the case of D_1_, ideally we would like to try to identify the peak of homozygous deletion and use the same method described for D_0_ to choose *E* and *β*. However, in practice this is almost never possible. The very small fraction of homozygous deletion relative to the large amount of background noise makes this procedure nearly impossible. So, for D_1_ we set the value of *E* such that it filters out the bulk of signal and only encodes what is extremely unlikely to be heterozygous signal. We set *E* for D_1_ to the 2.5^th^ percentile of the probe-level log_2_ ratio signal. This is an arbitrary cutoff and was selected again to be conservative. *β* for D_1_ is set equal to the value of *β* for D_0_ to keep the sigmoid function of D_1_ strictly less (more negative) than that of D_0_ so as to prevent doubly-encoding heterozygous signal. We impose a set of criteria to prevent the value of *E* for D_1_ from getting too small (negative). First, if it is less than 1.4× the value of *E* for D_0_, then we initialize *E* for D_1_ to the 1%-tile of the probe-level signal. If this is still greater than 1.4×E_0_ for D_0_, we set it equal to this value.

For A_1_, ideally we would like the sigmoid function to work as an integrator of amplification signal as described in the text. Heuristically, we chose the following parameters to accomplish this: *β* for A_1_ is fixed to a reduced value, equal to 1/(log 2). We also set *E* for A_1_ to 2× the value of *E* for A_0_ minus the value of the positive half-maximum of the diploid peak. This positions *E* for A_1_ on the supposed location of the +1 copy-number peak. As with D_1_, we incorporate a rule to bound *E* for A_1_ to be 1.4× the value of *E* for A_0_ or higher.

**C. Analytical derivation of error**

For the derivation of analytical error bars on the multi-component model *k* where *k* = {A_0_, A_1_, D_0_, and D_1_}, as well as for summary scores of total gain (A′) and loss (D′), we propagate the intrinsic error from original segmentation spanning a given region of the genome (illustrated for gain; similar implemented for loss). For reference, recall the scoring of log_2_ segmentation *a_ij_* is given by $S_{k(a_{\text{ij}})}$, the sigmoid function (Eq. 2, see text). Additionally, by example, the value of A_0_ is computed as the mean across samples and loci in a given breakpoint-defined region (UBP) (see text). Finally, recall the default combination of the multi-component model is the L_2_ norm (Eq. 3, see text). Given these implementations, and the set of segments from each sample spanning a given genomic region derived from the unified breakpoint profile (Figure 3A), we observe that *a_ij_* is the original segment mean *i* in sample *j* (the arithmetic mean of probe-level signal for markers encompassed by the segment, as defined by CBS segmentation), *s_ij_* is the standard deviation of the same segment, *n_ij_* is the number of markers belonging to the segment, and finally *N* is the count of tumors analyzed. Then we can define the square of the first derivative of the sigmoid-shaped function as:

For A_1_, the piece-wise change in its implementation (see text) is carried through to its error; the derivative in this case is of the form:

The theta function is implemented as describe in Eq. 2 in Methods. Subsequently, the individual-component error bars δ on, by example, A_0_ is:

This is repeated for each component of the multi-component model where ${DS}_{k(a_{\text{ij}})}$is individually parameterized with sample-specific *E_k_* and *β_k_* (see above). The final error bars on total gain are:

**II. Supplementary Results**

**A. Tumor Sequencing Project, Lung Adenocarcinoma**

We analyzed a large dataset of lung adenocarcinomas recently published under the aegis of the Tumor Sequencing Project [4]. To avoid variation or bias introduced by analyzing different tumor subsets, we collected the matching 371 tumors based on published quality control criteria (B.A. Weir, personal communication). We quantified and normalized this cohort as described in Methods. While the power of this dataset should prevent gross variability in results based on the inclusion or exclusion of small counts of tumors, several of the published lesions are low-frequency events (≤2%), so we applied no further filtering criteria. These tumors were parameterized as previously described in the text.

While a full and fine-scale analysis of the results is outside the scope of this report, we quantified the concordance between results in two ways. First, we collected all significant sub-arm length genomic alterations from the published study and mapped these, by coordinate boundaries, to markers on the array. These were then converted to regions of the unified breakpoint profile (UBP; n=14,402) to identify the genomic regions significantly altered from the original study. We examined the concordance between all significant regions of either amplification or deletion by comparing the published regions to those detected as significant by RAE at FDR ≤ 0.25 so as to match the published threshold (Table S4). The comparison of amplified, diploid, and deleted regions between the two methods was highly concordant (χ^2^ statistic > 1.32x10^4^). We additionally confirmed this estimate of concordance by calculating a second measure of correlation.

Because both methods assign a summary score to each region of the genome for both amplification and deletion (RAE, L_2_; GISTIC, G-score), we can directly compare these in regions of significant alteration. Therefore, we calculated the G-score in the following way. We first re-normalize individual-tumor segmentation by adding back to the segmentation mean of each marker the offset value that mode-centered the original distribution of segmentation (see Methods). We then subtract the median of segmentation in each tumor per the published approach [5]. This produces a segmentation profile on a scale similar to that of the original study. We then calculate the G-score for amplification and deletion as previously described for autosomal markers using published thresholds (±0.1) [5]. Scores are assigned to each region of the UBP as the median of the scores of individual markers belonging to a given region. We then computed the Kendall tau rank correlation coefficient between the G-score and L_2_ for amplification and deletion in the union of regions significantly altered in the published study and the RAE analysis (FDR ≤ 0.25). This reaffirms the concordance observed by the prior comparison (τ=0.86 and 0.77 for amplification and deletion respectively).

For the 31 focal amplification and deletions (41 including all supplementary events), we identified the regions of the UBP that partially or completely span the reported peak boundaries. We then identified which of each event was also statistically significant in the RAE analysis (either assessed from a null model of total gain or loss or from amplification (A_1_) or deletion (D_1_)-specific null models). 29 of 31 focal events from the published study were also significant in the RAE analysis (39 of 41 total reported events, 95%). The two focal alterations that did not reach statistical significance in RAE were the amplification of 2p15 and deletion of 7q11.22. Both of these are lower significance in the original study (published q-values=0.123 and 0.026 respectively). The former is a ~1.17mb amplification, and similar to the original study results, we also observe two tumors with substantial amplification of the locus, but this did not reach significance in our analysis (q-value=0.89). The latter deletion, a ~120kb loss of the *AUTS2* locus, is described as homozygous in three tumors and the original study investigators sequenced the gene and found no somatic mutations. The RAE analysis produced no homozygous deletion in *AUTS2* and 14 tumors with broad monoallelic loss. The lack of homozygosity is likely due to the stringency of the individual-tumor noise model (Table S5), as we observe two tumors with focal deletions in the segmentation data but whose log_2_ values approach, but do not exceed the value of *E_k_* for D_1_ in these tumors.

In summary, we found the agreement between the published results and those of RAE to be very good and reaffirm the emerging portrait of copy-number alterations in the lung adenocarcinoma genome. Of course, given the different approaches to normalization, down-stream data processing, and the methodological differences, we did not expect perfect agreement. The vast majority of anomalous events are likely due to these extraordinary factors unrelated to the core of either analytical approach. The balance of positive and negative differences is a repository of interesting events that highlight the value of investigating complex and dense genomic data with multiple approaches.

**B. Glioma**

We extended the comparison to a second tumor type, 141 recently described primary and secondary gliomas [5]. For RAE analysis, we downloaded the processed signal intensities provided by the original study authors (NCBI GEO; accession number GSE9635). We additionally downloaded updated mapping files for the Hind and Xba arrays of the Mapping 100K chip set (Affymetrix) and combined the individual arrays for each sample. We extracted the 141 tumors and 33 normal samples. We first ran a bootstrap quality control analysis of the 33 normal samples by first subtracting from log_2_ signal intensities the median of all normal samples at each marker. These were then segmented, normalized, and filtered with the previously described quality control criteria for normal samples used in germline filtering (see Methods). These criteria excluded 11 samples. Therefore, we normalized log_2_ signal intensities in the 141 tumors to a tumor/normal ratio by subtracting the median of log_2_ signal intensity in these 22 high-quality normal samples from each marker in each tumor. These tumors were then analyzed with RAE as described in the text.

For the 27 published regions of significant amplification or deletion (16 and 11 regions respectively), only the central genomic position of the detected peak was reported in lieu of start and end boundaries. So, we first converted these coordinate positions (reported in hg16) to the hg18 assembly of the human genome to match array annotation (UCSC; <http://genome.ucsc.edu/cgi-bin/hgLiftOver>). We then identified which of the reported peaks fell in regions of significant alteration in the RAE analysis by locating each region of the UBP for glioma (n=8,814) into which each reported peak falls (if the position fell between adjacent regions, the nearest region in genomic coordinates was chosen) (Table S4). This produced a 70.3% concordance rate for the reported peak positions between the two analyses. We then computed the correlation between summary scores calculated by each methodology in a manner similar to the lung adenocarcinoma dataset. However, because symmetric regions of significance could not be used due to the point-wise reporting of glioma regions, we instead calculated the Kendall tau rank correlation coefficient between L_2_ and G-scores for regions of significant amplification and deletion from RAE alone (FDR ≤ 0.01). Again, these were highly concordant and verify the previous result (τ=0.77 and 0.84 for amplification and deletion respectively).

Because we used signal intensities as processed and kindly provided by the original study authors, we took this opportunity to look in depth at each of the six amplifications and two deletions that were identified by the original study but sub-significantly altered in the RAE analysis. In all cases, alterations were 0.01 < q-value < 0.25 in the original study, so these are events at the margin of statistical significance. Also, given the threshold for significance in RAE of FDR≤1%, excluding these regions produced perfect concordance between methods. Additionally, there is a systematic difference in the levels at which events are considered altered between the two analyses. The original analysis used a symmetric global threshold of ±0.1 to determine aberrant copy number in log_2_ signal. On the other hand, the individual-tumor noise model used by RAE produces a value of *E_k_* for single-copy gain (A_0_) and hemizygous loss (D_0_) that is higher in nearly all tumors (n=138 of 141 for gain; n=134 of 141 for loss; Table S5, Figure S10). This difference in signal detection levels produces a global reduction in event frequency in the RAE analysis when compared with the original study results. This has a direct effect on assessment as each summary score is dominated by a frequency component and those scores are monotonic with p-value in both methods. In fact, the range of frequencies for significant amplifications reported in the original study is between 6 and 73% of tumors while RAE produces values between 5.7 and 51%. For deletions, these are from 17-71% and 11.3-61.7% respectively.

Six amplifications reported in the published study did not reach statistical significance in the RAE analysis. The 12q12.1 event spans 28 genes and whose peak was ~2mb from the locus encoding *KRAS*. This had a q-value=0.395 and was gained in 5.67% of tumors in the RAE analysis (down from the reported frequency of 11%). Of the eight tumors with gain at the locus, only one had a significant multi-copy amplification (RAE). The next locus was the amplification of 3q26.33 whose peak is ~526kb from the *PIK3CA* locus, had a q-value=0.684, and was gained in 7.8% of the cohort (11 tumors) as compared with the published frequency of 16%. None of the 11 tumors have what appears to be multi-copy amplification of the locus. The 8q24.12 peak, approximately ~6.7mb from the *MYC* locus has a q-value=0.544 and is gained in 8.51% of tumors (original frequency, 15%) for which two tumors (1.42%) have what appears to be significant amplifications. The 17q24.1 locus, amplified in 11% of tumors in the original study, has a q-value=0.724 in the RAE analysis. RAE considers the locus gained in 4.26% of tumors, and only one of these six tumors has substantial amplification. The last two amplifications, 6p21.1 and 2p24.3, were both gained in no more than 5.67% of tumors in the RAE analysis (7 and 11% in the original study) of which only the latter had tumors (n=2) with substantial amplifications (q-value=0.317).

The deletions of 4q34.3 and 11p15.4 were both sub-significant in the RAE analysis (q-value=0.394 and 0.74 respectively). The frequency of hemizygous loss at these loci were both smaller in the RAE analysis when compared to the published results (14.2 versus 17% and 13.5 versus 24% of tumors), likely owing to the noise model more stringently placing *E_k_*. Additionally, the frequencies of homozygous deletion at these loci were 1.42% and zero respectively.

Finally, in addition to the difference in detection levels and therefore frequencies of alteration, there are a subset of complex tumors whose mode-based normalization and median centering will produce very different alteration profiles (Figure S9). As the plot indicates, the median (for median-centering normalization) is in good agreement with the mode of the diploid peak in the majority of tumors. However, there is a subset of tumors in which the median does not represent the diploid component of the tumor, and scales tumors very differently than does shifting the distribution of segmentation by the mode of the putative diploid peak. Therefore this will affect the detection and recurrence levels of alterations for which those at the margins of significance are most sensitive.

**III. Supplementary Notes**

**Supplementary Note 1: Operational benefit of the unified breakpoint profile (UBP)**

The derived unified breakpoint profile (UBP) has a principal operational benefit. It produces an analysis on a count of regions slightly larger than segmentation of a single sample, but between one and three orders of magnitude smaller than the count of original markers on the array (~238,000). This helps remedy existing issues in multiple hypothesis correction. This problem is complicated in the context of whole-genome studies of DNA copy number when using either longstanding methods or those developed recently for DNA microarray analysis (transcriptional). The genome-wide association community has described this problem elegantly; of inappropriate corrections over multiple partially dependent tests [6]. In copy-number data, there is a tightly correlated structure; again the reason segmentation is so effective. This is because probes proximal to each other in the genome frequently share the same copy number. Thus, adjacency in genomic space likely produces a very similar if not identical copy number feature set. This implies that an executed statistical test is not truly independent as this class of methods expect, thus correcting over all markers equates to a considerable over-correction. The practical consequences of excessive over-correction are felt most acutely in analyses of rarer tumor types, when source material is limited, and the sample size is smaller than available for the more common epithelial tumors. In this context, the false discovery rate must be increased to levels that would otherwise tolerate too high a count of false positives to detect the full set significant events.

**Supplementary Note 2: Hyper-fragmentation during segmentation**

The problem of hyper-fragmentation of copy-number segmentation was investigated, and we find this is frequently the result of subtle striation patterns in the probe-level signal, likely due to local correlation effects between adjacent markers on higher density arrays. We discuss its manifestation in a single tumor (Figure S6). This tumor produced the highest count of segments over its genome of any single tumor analyzed thus far. It is an excellent example of the striation pattern that is unlikely the result of a biological phenomenon. When we plot probe-level data for a chromosome chosen at random, and then superimpose this with a spatially averaged version of the same by convolving with a Hamming window of 50, we see the de-noised trace indicates a periodicity in signal that is artifactual and likely the source of hyper-segmentation. Analysis of its power spectrum from the fast Fourier transform produces a similar periodic pattern at multiple frequencies (data not shown). Given the average distance between markers of 12.02kb (in this example, chromosome 1 after initial processing of raw data), a Hamming window of n=50 equates to a smoothing bandwidth of 601kb. Nevertheless, as mentioned in the primary text, samples harboring hyper-fragmentation are restored after derivation of the unified breakpoint profile, as they may still harbor the underlying disease copy number (Figure S7).

This problem can be artificially induced in samples lacking the periodicity in signal. CNAT implements a Gaussian smoothing operation during copy-number quantification that, given a user-defined window size whose default is 100kb, will smooth copy-number signal. This, however, violates assumptions made by, and generates a failure in, external CBS segmentation producing a very similar hyper-fragmented profile. Instead of artifactual periodicity, this convolved signal reduces the native and inherent variance between adjacent probes, causing segmentation to produce a change-point at positions of very small log-scale differences in neighboring copy number. We subsequently chose to reset this bandwidth parameter to zero, preventing smoothing of copy-number signal.

**Supplementary Note 3: Consequences of alternative permutation models**

We investigated the possibility of chromosome-specific permutation for the generation of a null distribution. We found, however, that per-chromosome permutation, that is, permuting regions and their values to random positions only on the chromosome to which it resides, is problematic for two reasons. The first is related to the space of detectable events. Per-chromosome permutation produces a null model suitable only for the detection of sub-chromosomal alterations. This is because, in the event of whole-chromosome or arm-length events, the permutation simply shuffles constantly and contiguously altered regions, independent of their position. While sub-chromosomal events are likely the most biologically compelling and least ambiguous with regard to experimental validation, larger events have been associated with both greater cancer susceptibility and poor clinical outcome in hematological malignancies [7,8]. The second reason stems from the pattern of overall aberrancy in a given tumor type. In the case of a lightly aberrant genome having a low count of focal gain and loss and lacking larger whole-arm-level events, the distributions of permuted gain or loss between chromosomes are stable and mostly overlap. Alternatively, in a highly aberrant cancer type, with multiple chromosomes having large and recurrent alterations, a far more frequent observation in human cancers from prior studies, will produce chromosome-specific distributions of permuted data whose mean aberrancy profile is different, higher, than that of quieter chromosomes in the same genome and disease type. This offset in distributions between chromosomes produces a suppression effect during event detection if the null distribution against which tumor regions are tested borrows permuted data between chromosomes. Consequently, we chose a null distribution derived from whole-genome permutation.

**IV. Supplementary References**

1. Bamshad MJ, Wooding S, Watkins WS, Ostler CT, Batzer MA, et al. (2003) Human population genetic structure and inference of group membership. Am J Hum Genet 72: 578-589.

2. Pritchard JK, Stephens M, Donnelly P (2000) Inference of population structure using multilocus genotype data. Genetics 155: 945-959.

3. Redon R, Ishikawa S, Fitch KR, Feuk L, Perry GH, et al. (2006) Global variation in copy number in the human genome. Nature 444: 444-454.

4. Weir BA, Woo MS, Getz G, Perner S, Ding L, et al. (2007) Characterizing the cancer genome in lung adenocarcinoma. Nature.

5. Beroukhim R, Getz G, Nghiemphu L, Barretina J, Hsueh T, et al. (2007) Assessing the significance of chromosomal aberrations in cancer: Methodology and application to glioma. Proc Natl Acad Sci U S A 104: 20007-20012.

6. (2007) Genome-wide association study of 14,000 cases of seven common diseases and 3,000 shared controls. Nature 447: 661-678.

7. Farag SS, Archer KJ, Mrozek K, Vardiman JW, Carroll AJ, et al. (2002) Isolated trisomy of chromosomes 8, 11, 13 and 21 is an adverse prognostic factor in adults with de novo acute myeloid leukemia: results from Cancer and Leukemia Group B 8461. Int J Oncol 21: 1041-1051.

8. Willenbucher RF, Aust DE, Chang CG, Zelman SJ, Ferrell LD, et al. (1999) Genomic instability is an early event during the progression pathway of ulcerative-colitis-related neoplasia. Am J Pathol 154: 1825-1830.
