# Supplementary material for: Functional Copy-Number Alterations in Cancer
Source: PLoS One. 2008 Sep 11;3(9):e3179. doi: 10.1371/journal.pone.0003179 (PMC2527508; doi:10.1371/journal.pone.0003179)
Supplement: Table S1 — Individuals of the HapMap collection included in the reference normal (0.31 MB DOC) [file pone.0003179.s002.doc]

**Table S1.** Individuals of the HapMap collection included in the reference normal

| **Sample** | **Identifier** | **Population** | **Gender** | **Family Identifier** | **Relationship** | **Partition** |
| --- | --- | --- | --- | --- | --- | --- |
| NA18524 | GSM117308 | Asian | Male | - | CHINA | A |
| NA18529 | GSM117310 | Asian | Female | - | CHINA | A |
| NA18537 | GSM117312 | Asian | Female | - | CHINA | A |
| NA18566 | GSM117325 | Asian | Female | - | CHINA | A |
| NA18570 | GSM117326 | Asian | Female | - | CHINA | A |
| NA18592 | GSM117334 | Asian | Female | - | CHINA | A |
| NA18603 | GSM117337 | Asian | Male | - | CHINA | A |
| NA18605 | GSM117338 | Asian | Male | - | CHINA | A |
| NA18620 | GSM117343 | Asian | Male | - | CHINA | A |
| NA18621 | GSM117344 | Asian | Male | - | CHINA | A |
| NA18635 | GSM117350 | Asian | Male | - | CHINA | A |
| NA18940 | GSM117405 | Asian | Male | - | JAPAN | A |
| NA18943 | GSM117407 | Asian | Male | - | JAPAN | A |
| NA18944 | GSM117408 | Asian | Male | - | JAPAN | A |
| NA18948 | GSM117411 | Asian | Male | - | JAPAN | A |
| NA18953 | GSM117415 | Asian | Male | - | JAPAN | A |
| NA18959 | GSM117417 | Asian | Male | - | JAPAN | A |
| NA18964 | GSM117420 | Asian | Female | - | JAPAN | A |
| NA18973 | GSM117429 | Asian | Female | - | JAPAN | A |
| NA18974 | GSM117430 | Asian | Male | - | JAPAN | A |
| NA18975 | GSM117431 | Asian | Female | - | JAPAN | A |
| NA18980 | GSM117434 | Asian | Female | - | JAPAN | A |
| NA18987 | GSM117436 | Asian | Female | - | JAPAN | A |
| NA18991 | GSM117438 | Asian | Female | - | JAPAN | A |
| NA18992 | GSM117439 | Asian | Female | - | JAPAN | A |
| NA18994 | GSM117440 | Asian | Male | - | JAPAN | A |
| NA19000 | GSM117445 | Asian | Male | - | JAPAN | A |
| NA18637 | GSM117551 | Asian | Male | - | CHINA | A |
| NA19003 | GSM117552 | Asian | Female | - | JAPAN | A |
| NA07345 | GSM117214 | Ceph | Female | 1345-13 | maternal grandmother | A |
| NA10847 | GSM117223 | Ceph | Female | 1334-2 | mother | A |
| NA12057 | GSM117254 | Ceph | Female | 1344-13 | paternal grandmother | A |
| NA12249 | GSM117265 | Ceph | Female | 1416-12 | paternal grandmother | A |
| NA12740 | GSM117270 | Ceph | Female | 1444-2 | mother | A |
| NA18504 | GSM117297 | Yoruba | Male | Y005-3 | father | A |
| NA18860 | GSM117395 | Yoruba | Male | Y012-1 | child | A |
| NA19132 | GSM117468 | Yoruba | Female | Y101-1 | child | A |
| NA19139 | GSM117471 | Yoruba | Male | Y043-1 | child | A |
| NA19223 | GSM117504 | Yoruba | Male | Y058-3 | father | A |
| NA19240 | GSM117509 | Yoruba | Female | Y117-1 | child | A |
| NA18526 | GSM117309 | Asian | Female | - | CHINA | B |
| NA18532 | GSM117311 | Asian | Female | - | CHINA | B |
| NA18540 | GSM117313 | Asian | Female | - | CHINA | B |
| NA18542 | GSM117314 | Asian | Female | - | CHINA | B |
| NA18545 | GSM117315 | Asian | Female | - | CHINA | B |
| NA18547 | GSM117316 | Asian | Female | - | CHINA | B |
| NA18550 | GSM117317 | Asian | Female | - | CHINA | B |
| NA18552 | GSM117318 | Asian | Female | - | CHINA | B |
| NA18555 | GSM117319 | Asian | Female | - | CHINA | B |
| NA18558 | GSM117320 | Asian | Male | - | CHINA | B |
| NA18561 | GSM117321 | Asian | Male | - | CHINA | B |
| NA18562 | GSM117322 | Asian | Male | - | CHINA | B |
| NA18563 | GSM117323 | Asian | Male | - | CHINA | B |
| NA18564 | GSM117324 | Asian | Female | - | CHINA | B |
| NA18571 | GSM117327 | Asian | Female | - | CHINA | B |
| NA18572 | GSM117328 | Asian | Male | - | CHINA | B |
| NA18573 | GSM117329 | Asian | Female | - | CHINA | B |
| NA18576 | GSM117330 | Asian | Female | - | CHINA | B |
| NA18577 | GSM117331 | Asian | Female | - | CHINA | B |
| NA18579 | GSM117332 | Asian | Female | - | CHINA | B |
| NA18582 | GSM117333 | Asian | Female | - | CHINA | B |
| NA18593 | GSM117335 | Asian | Female | - | CHINA | B |
| NA18594 | GSM117336 | Asian | Female | - | CHINA | B |
| NA18608 | GSM117339 | Asian | Male | - | CHINA | B |
| NA18609 | GSM117340 | Asian | Male | - | CHINA | B |
| NA18611 | GSM117341 | Asian | Male | - | CHINA | B |
| NA18612 | GSM117342 | Asian | Male | - | CHINA | B |
| NA18622 | GSM117345 | Asian | Male | - | CHINA | B |
| NA18623 | GSM117346 | Asian | Male | - | CHINA | B |
| NA18624 | GSM117347 | Asian | Male | - | CHINA | B |
| NA18632 | GSM117348 | Asian | Male | - | CHINA | B |
| NA18633 | GSM117349 | Asian | Male | - | CHINA | B |
| NA18636 | GSM117351 | Asian | Male | - | CHINA | B |
| NA18942 | GSM117406 | Asian | Female | - | JAPAN | B |
| NA18945 | GSM117409 | Asian | Male | - | JAPAN | B |
| NA18947 | GSM117410 | Asian | Female | - | JAPAN | B |
| NA18949 | GSM117412 | Asian | Female | - | JAPAN | B |
| NA18951 | GSM117413 | Asian | Female | - | JAPAN | B |
| NA18952 | GSM117414 | Asian | Male | - | JAPAN | B |
| NA18956 | GSM117416 | Asian | Female | - | JAPAN | B |
| NA18960 | GSM117418 | Asian | Male | - | JAPAN | B |
| NA18961 | GSM117419 | Asian | Male | - | JAPAN | B |
| NA18965 | GSM117421 | Asian | Male | - | JAPAN | B |
| NA18966 | GSM117422 | Asian | Male | - | JAPAN | B |
| NA18967 | GSM117423 | Asian | Male | - | JAPAN | B |
| NA18968 | GSM117424 | Asian | Female | - | JAPAN | B |
| NA18969 | GSM117425 | Asian | Female | - | JAPAN | B |
| NA18970 | GSM117426 | Asian | Male | - | JAPAN | B |
| NA18971 | GSM117427 | Asian | Male | - | JAPAN | B |
| NA18972 | GSM117428 | Asian | Female | - | JAPAN | B |
| NA18976 | GSM117432 | Asian | Female | - | JAPAN | B |
| NA18978 | GSM117433 | Asian | Female | - | JAPAN | B |
| NA18981 | GSM117435 | Asian | Female | - | JAPAN | B |
| NA18990 | GSM117437 | Asian | Male | - | JAPAN | B |
| NA18995 | GSM117441 | Asian | Male | - | JAPAN | B |
| NA18997 | GSM117442 | Asian | Female | - | JAPAN | B |
| NA18998 | GSM117443 | Asian | Female | - | JAPAN | B |
| NA18999 | GSM117444 | Asian | Female | - | JAPAN | B |
| NA19005 | GSM117448 | Asian | Male | - | JAPAN | B |
| NA19007 | GSM117449 | Asian | Male | - | JAPAN | B |
| NA19012 | GSM117450 | Asian | Male | - | JAPAN | B |
| NA06993 | GSM117203 | Ceph | Male | 1341-13 | maternal grandfather | B |
| NA07056 | GSM117213 | Ceph | Female | 1340-12 | maternalgrandmother | B |
| NA10830 | GSM117217 | Ceph | Male | 1408-1 | father | B |
| NA10859 | GSM117229 | Ceph | Female | 1347-2 | mother | B |
| NA11831 | GSM117237 | Ceph | Male | 1350-12 | maternal grandfather | B |
| NA11840 | GSM117240 | Ceph | Female | 1349-14 | maternal grandmother | B |
| NA11993 | GSM117244 | Ceph | Female | 1362-14 | paternal grandmother | B |
| NA12004 | GSM117248 | Ceph | Female | 1420-10 | paternal grandmother | B |
| NA12043 | GSM117251 | Ceph | Male | 1346-11 | paternal grandfather | B |
| NA12264 | GSM117266 | Ceph | Male | 1375-11 | maternal grandfather | B |
| NA12707 | GSM117267 | Ceph | Male | 1358-1 | father | B |
| NA12762 | GSM117277 | Ceph | Male | 1447-11 | maternal grandfather | B |
| NA12801 | GSM117279 | Ceph | Male | 1454-1 | father | B |
| NA12864 | GSM117285 | Ceph | Male | 1459-1 | father | B |
| NA12892 | GSM117550 | Ceph | Female | 1463-16 | maternal grandmother | B |
| NA18502 | GSM117295 | Yoruba | Female | Y004-2 | mother | B |
| NA18506 | GSM117299 | Yoruba | Male | Y009-1 | child | B |
| NA18516 | GSM117303 | Yoruba | Male | Y013-3 | father | B |
| NA18521 | GSM117305 | Yoruba | Male | Y016-1 | child | B |
| NA18853 | GSM117388 | Yoruba | Male | Y018-3 | father | B |
| NA18857 | GSM117392 | Yoruba | Male | Y023-1 | child | B |
| NA18863 | GSM117398 | Yoruba | Male | Y024-1 | child | B |
| NA18870 | GSM117399 | Yoruba | Female | Y017-2 | mother | B |
| NA18914 | GSM117404 | Yoruba | Male | Y028-1 | child | B |
| NA19094 | GSM117453 | Yoruba | Female | Y040-1 | child | B |
| NA19099 | GSM117455 | Yoruba | Female | Y105-2 | mother | B |
| NA19103 | GSM117459 | Yoruba | Male | Y042-1 | child | B |
| NA19116 | GSM117460 | Yoruba | Female | Y060-2 | mother | B |
| NA19127 | GSM117463 | Yoruba | Female | Y077-2 | mother | B |
| NA19141 | GSM117473 | Yoruba | Male | Y071-3 | father | B |
| NA19145 | GSM117477 | Yoruba | Male | Y074-1 | child | B |
| NA19153 | GSM117479 | Yoruba | Male | Y072-3 | father | B |
| NA19161 | GSM117483 | Yoruba | Male | Y056-1 | child | B |
| NA19173 | GSM117486 | Yoruba | Male | Y047-1 | child | B |
| NA19193 | GSM117488 | Yoruba | Female | Y112-2 | mother | B |
| NA19202 | GSM117492 | Yoruba | Female | Y045-1 | child | B |
| NA19205 | GSM117495 | Yoruba | Male | Y048-1 | child | B |
| NA19208 | GSM117498 | Yoruba | Male | Y051-1 | child | B |
| NA19211 | GSM117501 | Yoruba | Male | Y050-1 | child | B |
