# Supplementary material for: Functional Copy-Number Alterations in Cancer
Source: PLoS One. 2008 Sep 11;3(9):e3179. doi: 10.1371/journal.pone.0003179 (PMC2527508; doi:10.1371/journal.pone.0003179)
Supplement: Table S2 — Additional genomic gain/amplification in pleomorphic liposarcoma (0.10 MB DOC) [file pone.0003179.s003.doc]

**Table S2.** Additional genomic gain/amplification in pleomorphic liposarcoma

|  |  |  |  | **Number of genes‡** | **Genetic elements of interest** |  | **Spanning known structural variation (CNV)§#** | | |  |  |
| --- | --- | --- | --- | --- | --- | --- | --- | --- | --- | --- | --- |
| **Locus** | **Region (peak)*** | | **Q-value** |  |  |  | **Gain** | **Loss** | **Unknown** |  | **Notes** |
| 1p32.3 | 53592377-53845315 | | 5.70E-03 | 2 | *GLIS1* |  | - | - | 43.8 |  |  |
| 1p32.3-p32.2 | 55515432-58444487 | | 3.83E-05 | 7 | *PPAP2B,PRKAA2,DAB1* |  | 4.1 (9) | 4.1 (3) | - |  | *Near common fragile site* |
| 1p31.3 | 60338189-61131213 | | 8.75E-03 | 0 | - |  | - | - | - |  | *Multiple ESTs* |
| 1p31.1 |  | 77218238-77220772 | 1.51E-05 | 1 | *ST6GALNAC5* |  | 100 (34) | - | - |  | *Known polymorphism* |
| 1p31.1 | 79045061-83975654 | | 3.34E-05 | 1 | - |  | 14.6 (3.3) | 45.3 (1.2) | 13.8 |  |  |
| 1p22.3 | 87339231-87644753 | | 6.26E-03 | 1 | - |  | - | 0.9 (1) | 0.9 |  |  |
| 1p21.1-p13.3 | 106302215-107095587 | | 6.26E-03 | 0 | - |  | 1.2 (1) | 8.5 (1) | 15.3 |  | *Likely polymorphism* |
| 1q21.3 | 148401888-150244695 | | 6.09E-04 | 57 | *RORC,S100A8* |  | 18.1 (10) | 13.4 (1.6) | 23.3 |  |  |
| 5p15.31 | 7368552-7580982 | | 4.52E-03 | 1 | - |  | 69.9 (2) | - | - |  |  |
| 5p15.31 | 7596807-7942216 | | 4.33E-03 | 3 | *MTRR* |  | - | 1.6 (1) | - |  |  |
| 5p15.31 | 9308046-9495192 | | 4.47E-03 | 1 | - |  | - | - | - |  |  |
| 5p15.2 | 10035966-10037591 | | 5.64E-03 | 0 | - |  | - | 100 (1) | 100 |  | *Known polymorphism* |
| 5p15.1-p13.3 | 15788014-31602359 | | 2.63E-05 | 13 | *CDH6,CDH9,CDH10,DROSHA* |  | 9 (5.1) | 14 (4.1) | 12.9 |  |  |
| 5p13.3 |  | 29514081-31406275 | 2.63E-05 | 1 | *CDH6* |  | - | 1.3 (2) | 3.9 |  |  |
| 5p13.3 | 31669008-31713955 | | 5.37E-03 | 0 | - |  | - | - | - |  | *Multiple ESTs* |
| 6p21.32 | 33163924-33168818 | | 8.70E-03 | 0 | - |  | - | - | - |  | *Near HLA-DPB1* |
| 7p15.3 | 22083397-22243274 | | 3.31E-03 | 1 | *RAPGEF5* |  | - | - | - |  |  |
| 7p14.3-p14.1 | 34400418-37787156 | | 3.78E-03 | 14 | *EPDR1* |  | 14.5 (4) | 3.3 (1.6) | 16.4 |  |  |
| 7p12.3 | 47118242-47626738 | | 2.29E-03 | 3 | *TNS3* |  | - | 7.4 (2) | - |  |  |
| 7p12.3 | 48759008-49337116 | | 1.94E-03 | 0 | - |  | 33.6 (8) | 0.3 (1) | - |  | *Multiple ESTs and recombination hotspots* |
| 7p12.3-p12.2 | 49701581-49870339 | | 3.52E-03 | 2 | - |  | - | - | - |  |  |
| 7q11.21 | 63056934-63284920 | | 1.02E-03 | 0 | - |  | 57.5 (1) | - | - |  | *Segmental duplications* |
| 19p12 | 23144864-23321338 | | 4.79E-03 | 0 | - |  | 100 (1.8) | 100 (2.5) | 100 |  | *Known polymorphism* |
| 19p12-q13.11 | 24161928-40254153 | | - | - | - |  | - | - | - |  |  |
| 19q12 |  | 36956094-37056038 | <5.22E-06 | 0 | - |  | - | - | - |  |  |
|  |  |  |  |  |  |  |  |  |  |  |  |
| * Genomic boundaries detected as peaks within regions of contiguous alteration are indented (see Table 1) | | | | | | | | | | | |
| ‡ RefSeq (hg17); in parentheses, human microRNAs | | | | | | | | | | | |
| § Locus of alteration spanning known population CNV (see Methods), percent genomic coverage; in parentheses, mean sample count | | | | | | | | | | | |
| # Unknown: ambiguous direction of copy number variant | | | | | | | | | | | |

Genomic gains not listed in Table 1 of the primary text. Regions of high array density generate local correlations in signal between neighboring SNPs when not separated by StyI restriction sites, producing sub-resolution event size and statistical significance. These are considered erroneous and excluded.
