# Supplementary material for: Functional Copy-Number Alterations in Cancer
Source: PLoS One. 2008 Sep 11;3(9):e3179. doi: 10.1371/journal.pone.0003179 (PMC2527508; doi:10.1371/journal.pone.0003179)
Supplement: Table S3 — Additional genomic loss/deletion in pleomorphic liposarcoma (0.13 MB DOC) [file pone.0003179.s004.doc]

**Table S3.** Additional genomic loss/deletion in pleomorphic liposarcoma

|  |  |  |  |  |  |  | **Spanning known structural variation (CNV)§#** | | |  |  |
| --- | --- | --- | --- | --- | --- | --- | --- | --- | --- | --- | --- |
|  |  |  |  | **Number of genes‡** | **Genetic elements of interest** |  |  |  |  |  |  |
| **Locus** | **Region (peak)*** | | **Q-value** |  |  |  | **Gain** | **Loss** | **Unknown** |  | **Notes** |
| 1q42.2 | 227410960-227624922 | | 4.51E-03 | 4 | *TRIM67* |  | - | - | - |  |  |
| 1q43 | 233706993-233708730 | | 4.51E-03 | 1 | - |  | - | - | - |  |  |
| 1q43 | 238565609-238863653 | | 4.51E-03 | 1 | *PLD5* |  | 31.3 (2) | - | - |  |  |
| 2q37.1 | 231431588-234120935 | | 2.02E-03 | 34 (1) | *SAG,PTMA,COPS7B* |  | 5.2 (1) | 0.2 (1) | 18.4 |  |  |
| 2q37.2-q37.3 | 236203824-237192658 | | 4.94E-03 | 4 | *GBX2* |  | 21.3 (1) | 25.4 (1) | - |  |  |
| 2q37.3 | 240691509-241616408 | | 5.53E-03 | 14 (1) | *GPC1,DUSP28* |  | 100 (2) | 12 (1) | 24.2 |  |  |
| 2q37.3 | 241699540-242730382 | | 9.91E-04 | 17 | *FARP2,STK25,BOK,DTYMK* |  | 100 (1.7) | 55.6 (14.1) | 18.7 |  | *71.3kb germ-line variant in two normal samples, non-genic at telomeric end of the alteration* |
|  |  |  |  |  |  |  |  |  |  |  |  |
| 4p14 | 40191691-40563778 | | 1.28E-03 | 2 | - |  | 9.6 (2) | 0.9 (1) | 50.5 |  |  |
| 10p14-p13 | 11895605-12375594 | | 3.45E-03 | 6 | - |  | 69 (1.5) | - | - |  |  |
| 10p11.1-q11.21 | 38996885-42002139 | | 7.47E-03 | 0 | - |  | 12.2 (1.7) | 7.3 (3.1) | 6.9 |  |  |
| 10q21.3-q22.1 |  | 69705227-70622139 | 2.65E-05 | 14 | - |  | 0.6 (2) | 0.5 (2.5) | 18.9 |  | *Likely polymorphism, germline variant in 3 normal samples at 100% coverage* |
|  |  |  |  |  |  |  |  |  |  |  |  |
| 10q22.1 | 71122291-71700582 | | 3.78E-03 | 7 | *AIFM2* |  | 100 (15) | 100 (2) | - |  |  |
| 10q22.1 | 71779052-72385871 | | 3.78E-03 | 9 | *NODAL* |  | 72.1 (15) | 72.1 (1.5) | - |  |  |
| 10q23.31 | 89770130-90830274 | | 4.51E-03 | 8 | *FAS (near PTEN)* |  | 17.3 (3) | - | 0.5 |  |  |
| 10q23.33 | 95818543-96454720 | | 8.85E-03 | 4 | *PLCE1* |  | - | - | - |  |  |
| 12p13.2 | 10248041-10293894 | | 4.94E-03 | 1 | - |  | - | - | - |  |  |
| 12q24.23-q24.31 | 119225485-119862925 | | 4.94E-03 | 15 | *RNF10* |  | - | - | - |  | *Likely polymorphism, germline variant in 4 normal samples at 100% coverage* |
|  |  |  |  |  |  |  |  |  |  |  |  |
| 12q24.31 | 121153099-122631895 | | 4.94E-03 | 32 | - |  | 46.4 (1.5) | 13.4 (2) | - |  | *Same as above* |
| 13q14.2-q14.3 | 47620620-50574663 | | <5.74E-06 | 20 (2) | *RCBTB2,RB1* |  | 6.7 (7.2) | 33.4 (1.2) | 6.4 |  |  |
| 14q24.2-q24.3 | 72435913-73267706 | | 2.12E-04 | 15 | *PSEN1,NUMB,PNMA1* |  | 6.8 (2.5) | 6.8 (42) | 20 |  |  |
| 15q11.2 | 18753439-19207088 | | 4.94E-03 | 1 | - |  | 100 (46.4) | 100 (18.8) | 100 |  | *Known polymorphism, germline variant in 5 normal samples at 100% coverage* |
|  |  |  |  |  |  |  |  |  |  |  |  |
| 15q11.2 | 22216693-22629037 | | 6.40E-03 | 2 | - |  | 41.3 (26) | 48.1 (3.6) | 57.1 |  |  |
| 16q22.1 | 65777958-65787923 | | 8.64E-03 | 2 | *E2F4* |  | - | - | - |  |  |
| 16q22.1 | 66551420-66673519 | | 4.99E-03 | 5 | - |  | - | - | - |  |  |
| 16q24.3 | 88363022-88684276 | | 1.27E-04 | 11 | *FANCA* |  | 100 (20.5) | 60.8 (2.7) | 30.3 |  | *Known polymorphism, weak and low-frequency germline event with full coverage* |
|  |  |  |  |  |  |  |  |  |  |  |  |
| 17p13.3 | 6888-1362660 | | 1.27E-04 | 16 | *SKIP,GEMIN4,ABR* |  | 99.1 (1.7) | 7 (10.3) | 39.6 |  | *Likely polymorphism, low-frequency germline variants, 2 normal samples spanning 60.8% of the locus* |
|  |  |  |  |  |  |  |  |  |  |  |  |
| 17p13.3-p13.2 | 2751237-4797122 | | 1.09E-03 | 54 | *MINK1,CXCL16* |  | 6.6 (1.5) | 46.9 (4.3) | - |  | *Likely polymorphism, low-frequency germline variants, 2 normal samples spanning 58.8% of the locus* |
|  |  |  |  |  |  |  |  |  |  |  |  |
| 17p13.2-p13.1 | 6140525-6666473 | | 8.63E-03 | 10 | *XAF1* |  | 4.3 (1) | - | - |  |  |
| 22q13.33 | 48299314-49316351 | | 4.94E-03 | 29 | *MAPK11* |  | 67.3 (3) | 10.1 (4) | 8.6 |  |  |
|  |  |  |  |  |  |  |  |  |  |  |  |
| * Genomic boundaries detected as peaks within regions of contiguous alteration are indented (see Table 1) | | | | | | | | | | | |
| ‡ RefSeq (hg17); in parentheses, human microRNAs | | | | | | | | | | | |
| § Locus of alteration spanning known population CNV (see Methods), percent genomic coverage; in parentheses, mean sample count | | | | | | | | | | | |
| # Unknown: ambiguous direction of copy number variant | | | | | | | | | | | |

Genomic losses not listed in Table 1 in the primary text. Regions of high array density generate local correlations in signal between neighboring SNPs when not separated by StyI restriction sites, producing sub-resolution event size and statistical significance. These are considered erroneous and excluded.
