# Supplementary material for: Functional Copy-Number Alterations in Cancer
Source: PLoS One. 2008 Sep 11;3(9):e3179. doi: 10.1371/journal.pone.0003179 (PMC2527508; doi:10.1371/journal.pone.0003179)
Supplement: Table S4 — Comparison of significant events in the lung and glioma datasets. (0.05 MB DOC) [file pone.0003179.s005.doc]

**Table S4: Comparison of significant events in the lung and glioma datasets.**

| **A.)** |  |  |  |  |  |  |  |  |  |
| --- | --- | --- | --- | --- | --- | --- | --- | --- | --- |
|  |  |  |  |  |  |  |  |  |  |
| Amplification | 10 | - | 0 |  |  |  |  |  |  |
| Diploid | 6 | - | 2 |  |  |  |  |  |  |
| Deletion | 0 | - | 9 |  |  |  |  |  |  |
|  | Amplification | Diploid | Deletion |  |  |  |  |  |  |
|  |  |  |  |  |  |  |  |  |  |
| **B.)** |  |  |  |  | **C.)** |  |  |  |  |
|  |  |  |  |  |  |  |  |  |  |
| Amplification | 23 | - | 0 |  | Amplification | 65.9 | 13.4 | 0.41* |  |
| Diploid | 1 | - | 1 |  | Diploid | 20.7 | - | 13.7 |  |
| Deletion | 0 | - | 6 |  | Deletion | 0.98* | 13.2 | 73.1 |  |
|  | Amplification | Diploid | Deletion |  |  | Amplification | Diploid | Deletion |  |

Shown are tables (3x3) of amplification, deletion, and diploid events in either the lung or glioma datasets. Along the vertical are RAE assignments and along the horizontal are GISTIC assignments. **A,** The RAE status assigned to each reported focal event in glioma. **B,** The RAE status of each reported focal event in lung adenocarcinoma. **C,** The percentage of total genomic regions (n=14,402, UBP) and their status in RAE compared to reported regions.
