# Supplementary material for: Functional Copy-Number Alterations in Cancer
Source: PLoS One. 2008 Sep 11;3(9):e3179. doi: 10.1371/journal.pone.0003179 (PMC2527508; doi:10.1371/journal.pone.0003179)
Supplement: Table S5 — Values of Ek for lung and glioma datasets (0.04 MB DOC) [file pone.0003179.s006.doc]

**Table S5: Values of *E_k_* for lung and glioma datasets.**

|  | TSP (n=371) | |  | Glioma (n=141) | |
| --- | --- | --- | --- | --- | --- |
|  | **A0 (A1)** | **D0 (D1)** |  | **A0 (A1)** | **D0 (D1)** |
| **min** | 0.094 (0.164) | -0.094 (-0.473) |  | 0.094 (0.164) | -0.079 (-0.538) |
| **median** | 0.133 (0.233) | -0.134 (-0.726) |  | 0.182 (0.32) | -0.184 (-0.915) |
| **mean** | 0.155 (0.266) | -0.173 (-0.727) |  | 0.201 (0.352) | -0.205 (-0.92) |
| **max** | 0.528 (0.8729) | -0.772 (-1.08) |  | 0.599 (0.945) | -1.07 (-1.489) |

Shown are the minimum, maximum, mean, and median values of individual-tumor *E_k_* parameters in the lung and glioma datasets for each of four detectors (A_0_, A_1_, D_0_, and D_1_). In contrast, symmetric log_2_ thresholds of ±0.1 were used in the original studies.
