# Supplementary figures and images for: Functional Copy-Number Alterations in Cancer
Source: PLoS One. 2008 Sep 11;3(9):e3179. doi: 10.1371/journal.pone.0003179 (PMC2527508; doi:10.1371/journal.pone.0003179)

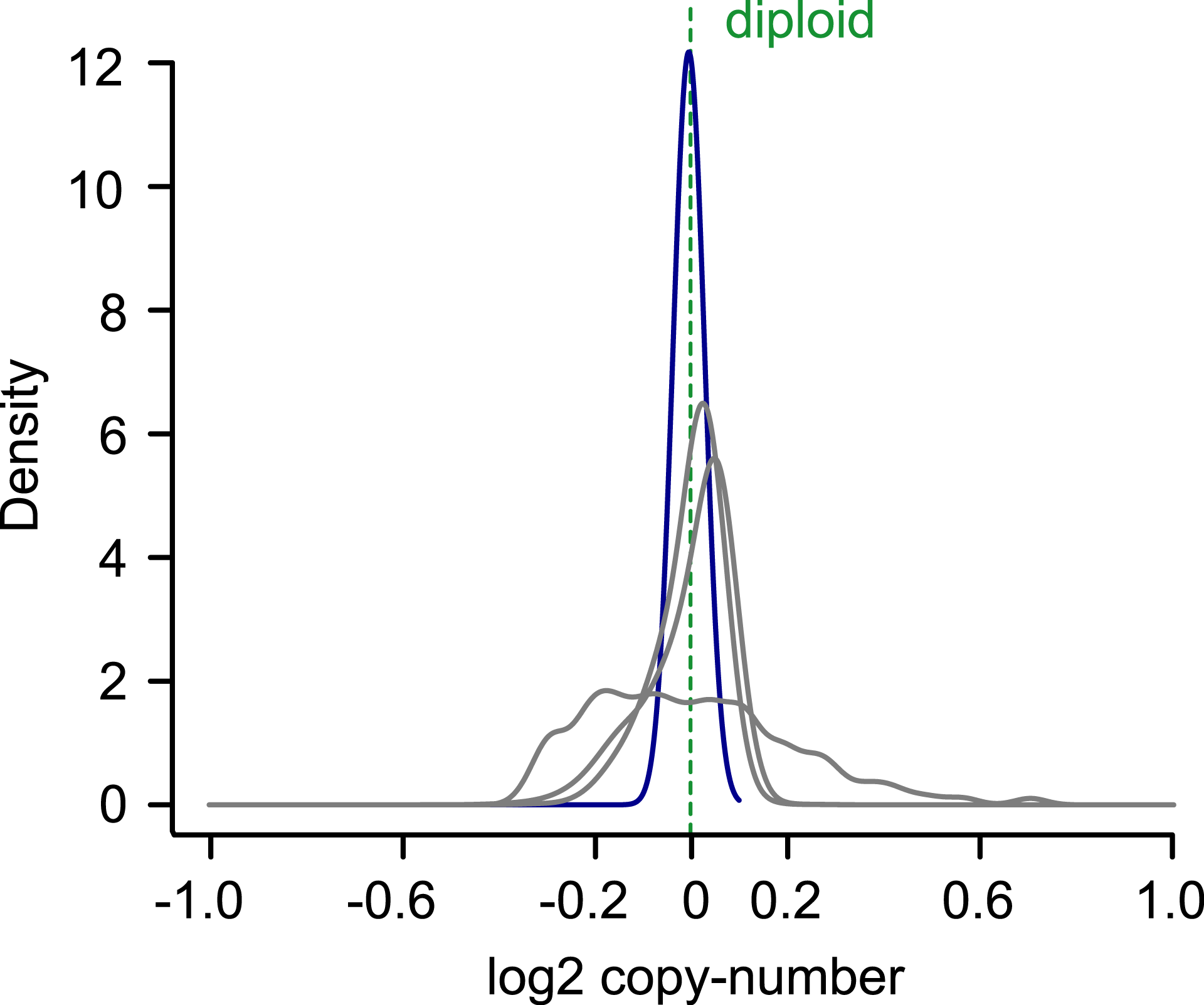

Supplement: Figure S1 — Diploid copy number between patient-matched non-tumor DNA and HapMap reference normal. The distribution of segment means weighted by their size for a randomly chosen HapMap individual from the copy-called partition (HapMap.B) of the reference normal (blue) and three tumor-matched normal samples (gray). The tight and symmetric diploid peak of the HapMap individual is juxtaposed to poorly behaved distributions of patient-matched normal DNA copy-number data. (0.33 MB TIF) [file pone.0003179.s007.tif]

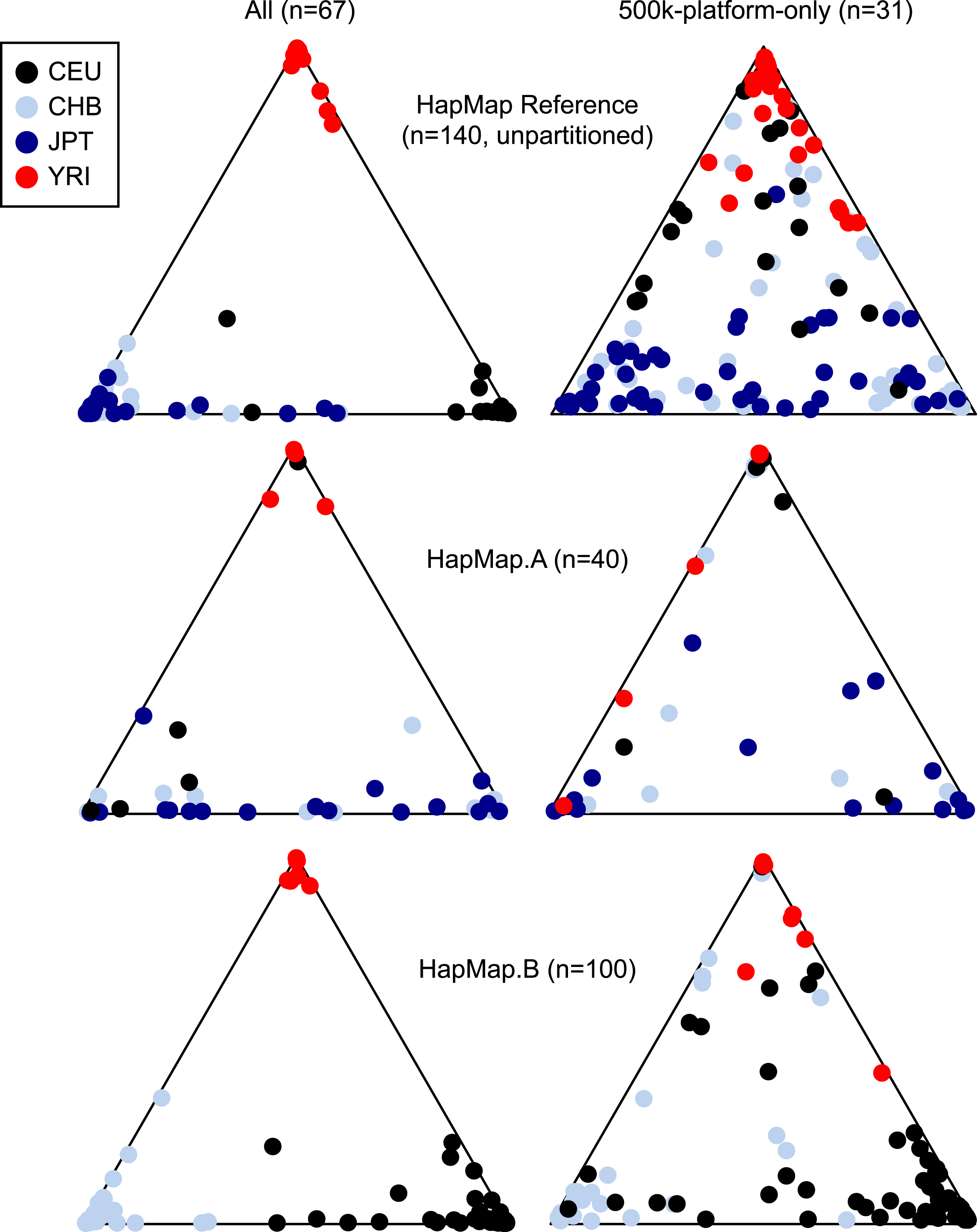

Supplement: Figure S2 — Confirming population genetic structure of HapMap reference normal. Population clustering of n = 140, 40, and 100 HapMap individuals from the partitions of the reference normal, assuming three ancestral populations (k = 3; triangle plots). Clustering is based on the 67 non-redundant biallelic CNVs from Redon et al. [ref. S3] and repeated on only those 31 genotypes derived from the Affymetrix early access platform (as indicated). (0.85 MB TIF) [file pone.0003179.s008.tif]

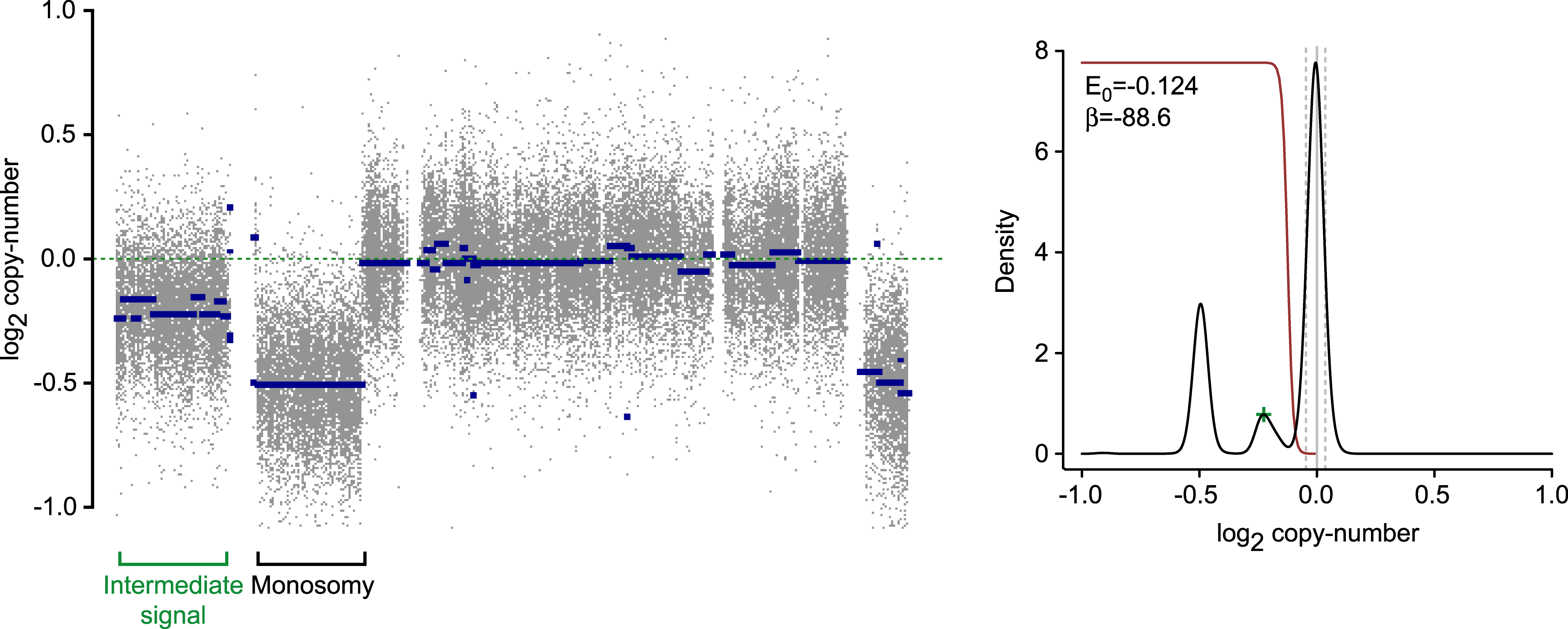

Supplement: Figure S3 — Detecting an intermediate phenotype in copy number. In a single tumor, monosomic chromosome 15 is adjacent to intermediate signal on chromosome 14 (probe-level data in gray, segmentation in blue). At right is the density distribution of autosomal segmentation weighted by event size (same annotation as in Figure S5). The peak representing the medial loss signal (green plus) may arise from either multiple tumor-cell populations in the DNA isolate, or allele-specific copy number (dark red sigmoid; D0 parameterized with indicated values of E and β). (0.62 MB TIF) [file pone.0003179.s009.tif]

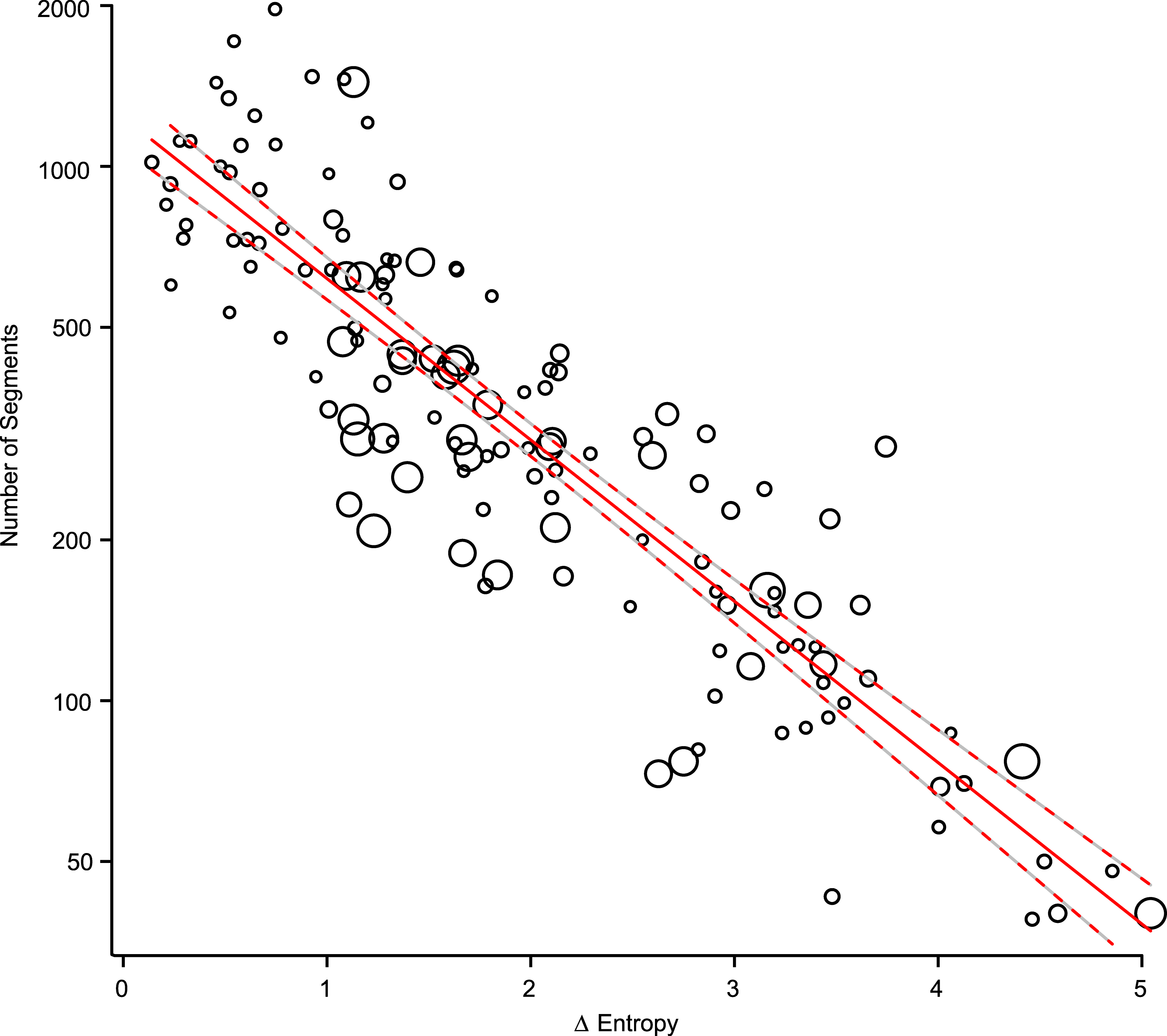

Supplement: Figure S4 — Relationship between probe-level and segmentation noise. The two very different types of noise associated with array-based copy number data. We can view the segmentation algorithm as a de-noising step that attempts to remove noise in probe-level measurements to accurately estimate the local copy number. Here, we measure the efficiency of this noise reduction by calculating the change in entropy from probe-level data to the entropy for segmentation values (means). To precisely define the entropy, we first compute the probability density histograms of both the probe-level and segmentation data using the R density function with a fixed bandwidth, limits (from −1 to 1 in log2 ratio units), and a fixed number of bins (2048). The entropy is then defined as shannon entropy where pi is the probability for each of the 2048 bins. When segmentation works properly, we observe a large change in entropy [where ΔS = S(r)−S(m)] from the probe-level data (r) to the segmentation values (m). However, the phenomenon of hyper-segmentation (which we have observed in many tumors; Methods S1, Figure S6) occurs when the segmentation algorithm generates a large number segments (far larger a number than are likely to be in the real data). As one would expect there is a simple relationship between this over-segmentation and the reduction in the change in entropy. However, these poorly behaved tumors, as measured by over-segmentation, seem to be uncorrelated with the low-level noise in their probe-level data as measured by the derivative noise (DN; see Methods). The derivative noise is represented by the size of the circles, where the radius in proportional to the DN. Notice that DN is roughly uniformly scattered along the ΔS x-axis. (0.72 MB TIF) [file pone.0003179.s010.tif]

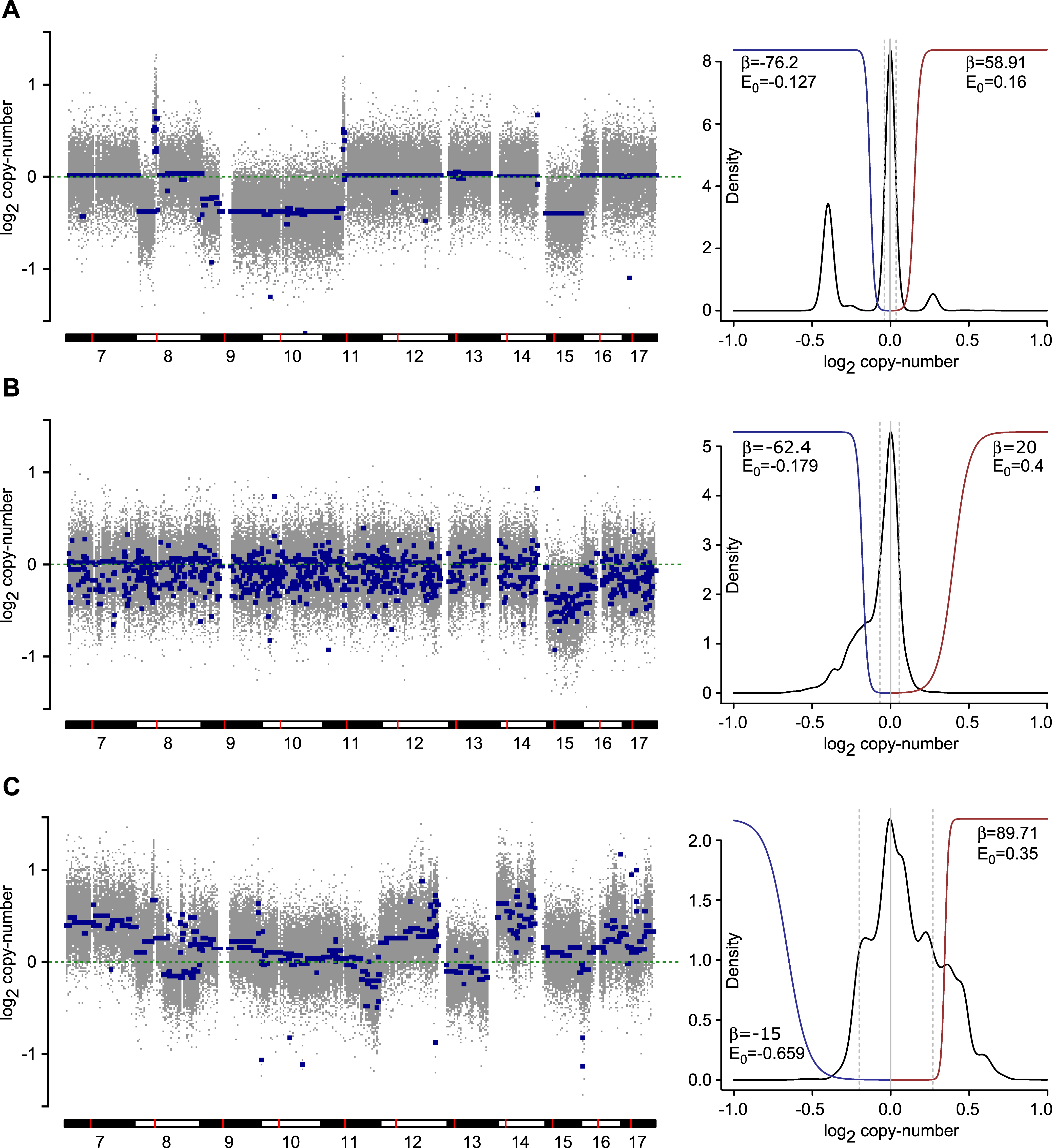

Supplement: Figure S5 — Diversity and heterogeneity of tumor profile. At left is probe-level (gray) and segmentation data (blue) for 11 chromosomes (indicated, centromere in red). At right is the density distribution of segmentation means weighted by their size (22 autosomes). For each, the sigmoid detecting single-copy gain (A0) and hemizygous loss (D0) are indicated (red and blue respectively, parameters labeled). The diploid peak is identified at zero in log2 copy-number (gray line) and the half-maximum values of the diploid peak are identified (dotted gray lines). (a) Tumor with a symmetric and well-behaved diploid peak as well as smaller peaks of detectable signal (at left, on chromosomes 8p, 9, 10, 11, and 15). (b) A hyper-segmented tumor with asymmetry in the diploid peak exclusively in deletion, challenging the choice of E for single-copy loss (D0). Nested signal can be detected in the form of whole-chromosome loss of chr15, an event detected in panel A as well. (c) A tumor having a highly complex and reduced-quality segmentation profile, gross asymmetry in both gain and loss, an ill-defined diploid peak, and lacks discernible features for the selection of parameters for its transformation in either gain or loss. (1.69 MB TIF) [file pone.0003179.s011.tif]

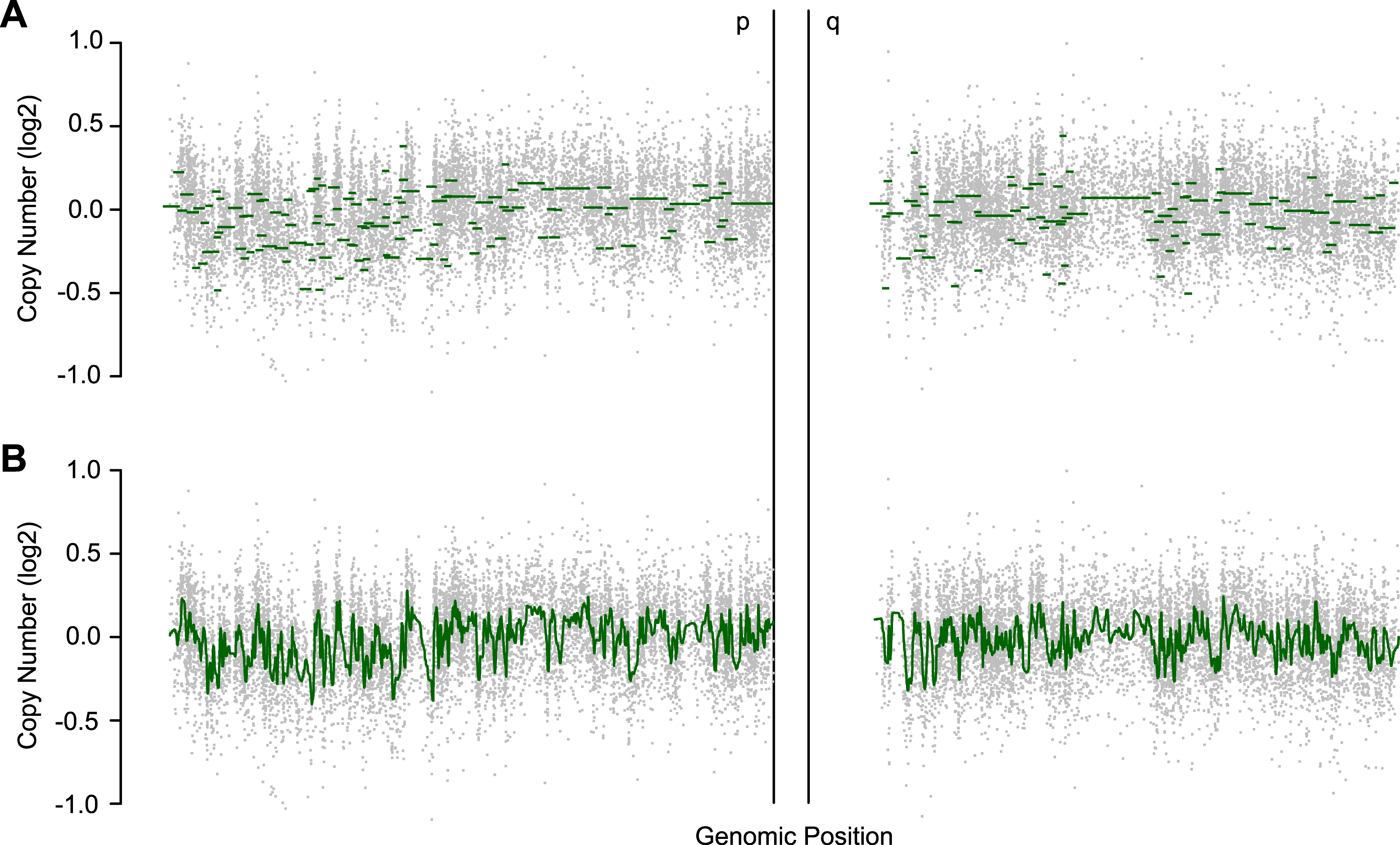

Supplement: Figure S6 — Hyper-fragmentation of copy number segmentation. (a) Normalized probe-level (gray) and segmentation (green) of chromosome 1 in a single tumor displaying a hyper-fragmentation pattern. (b) The same probe-level signal as in panel A, superimposed with a spatially averaged (bandwidth of ∼601 kb) version. Convolved trace indicates a non-disease related periodicity in signal likely the source of hyper-segmentation (Methods S1). (0.94 MB TIF) [file pone.0003179.s012.tif]

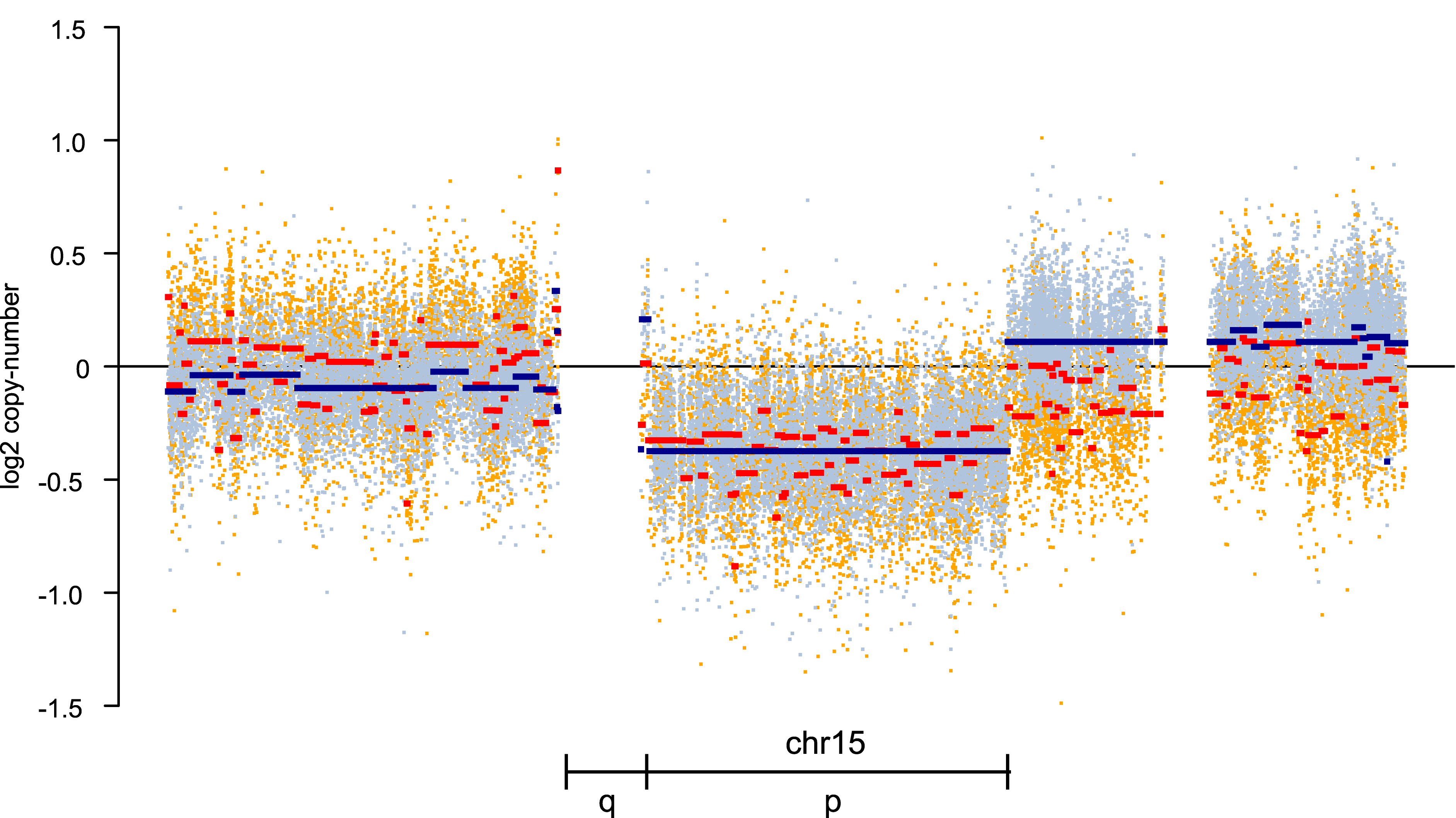

Supplement: Figure S7 — Varying segmentation quality between tumors does not preclude detection. Here, three chromosomes (14, 15, and 16) in two tumors, probe-level (orange and light blue) and segmentation data (red and dark blue, respectively). A high-quality segmentation result (dark blue) identifies monoallelic loss of the q-arm of chromosome 15. The lower-quality hyper-segmentation (red) also includes the 15q loss (highlighted). This motivates the conditional inclusion of both samples during scoring and assessment, but not the latter during UBP derivation. (0.93 MB TIF) [file pone.0003179.s013.tif]

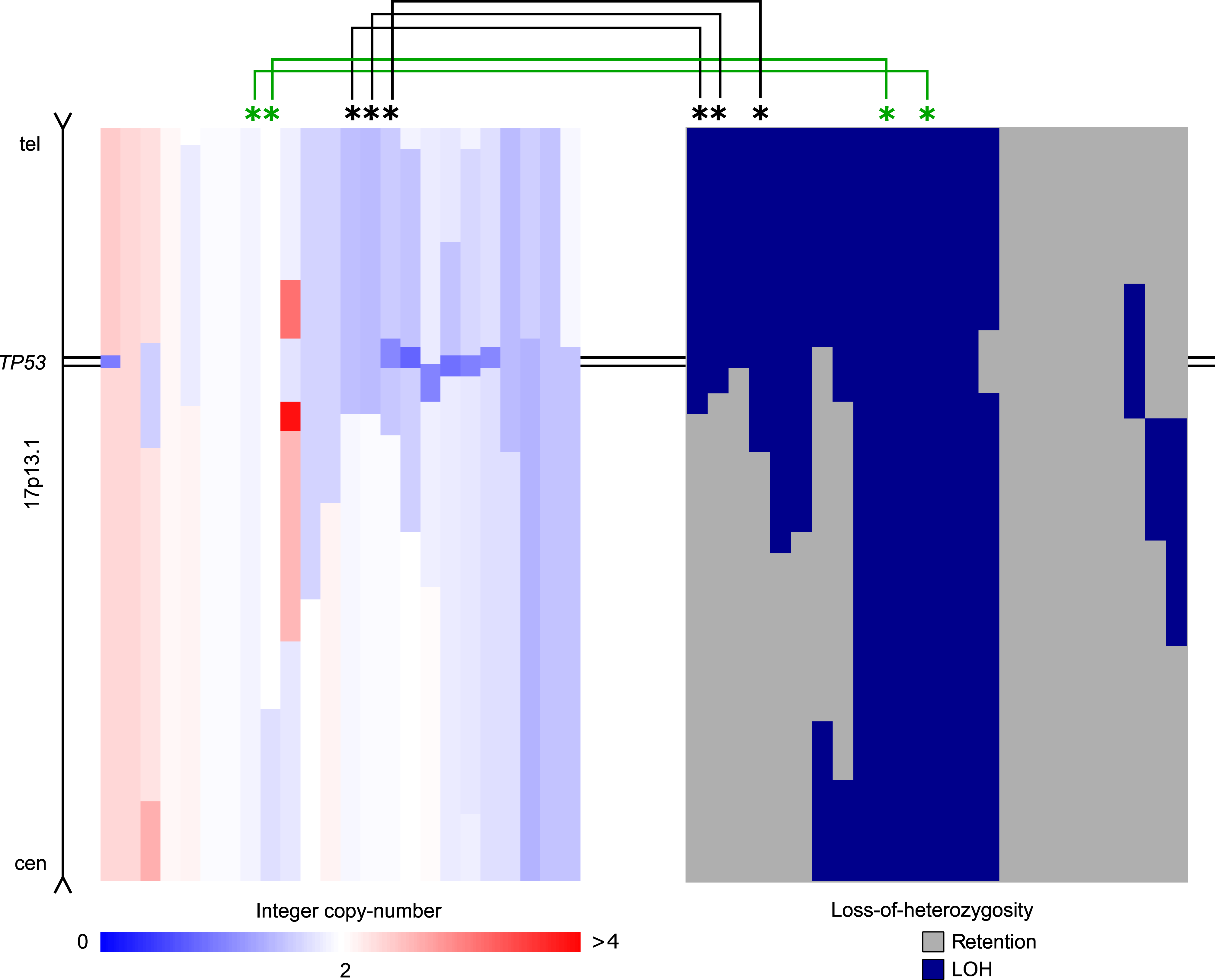

Supplement: Figure S8 — Genomic deletion and observed loss-of-heterozygosity for the p53 locus. Independent hierarchical clustering of copy number (segmentation, left) and LOH (paired, right) for 2.2 mb of 17p13.1 (columns are samples, rows are markers) indicates two patterns of alteration in pleomorphic liposarcomas. Deletion-associated LOH for p53 in three tumors with either broad or focal deletion (lines connect corresponding samples; black), and copy-neutral LOH (green). (2.93 MB TIF) [file pone.0003179.s014.tif]

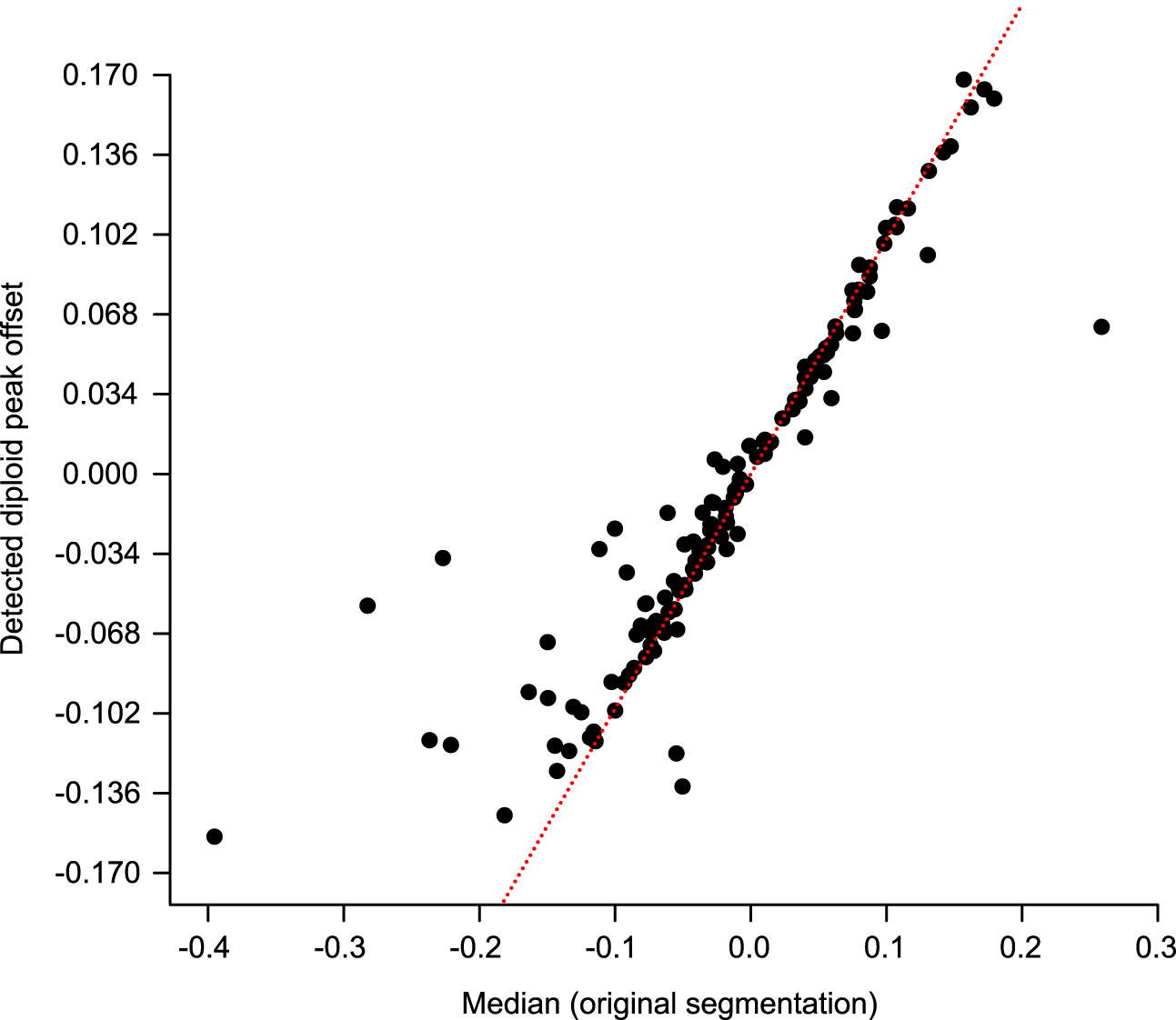

Supplement: Figure S9 — The affect of normalization on segmentation profiles. Here, the median of original segmentation of each glioma tumor (x-axis) and the distance (offset) of the mode of the diploid peak from log2 = 0 are plotted. While the median is a reasonable approximation for the diploid feature of most tumors, in a subset of tumors, the mode of the diploid peak and the median of un-normalized segmentation are substantially different. (0.26 MB TIF) [file pone.0003179.s015.tif]

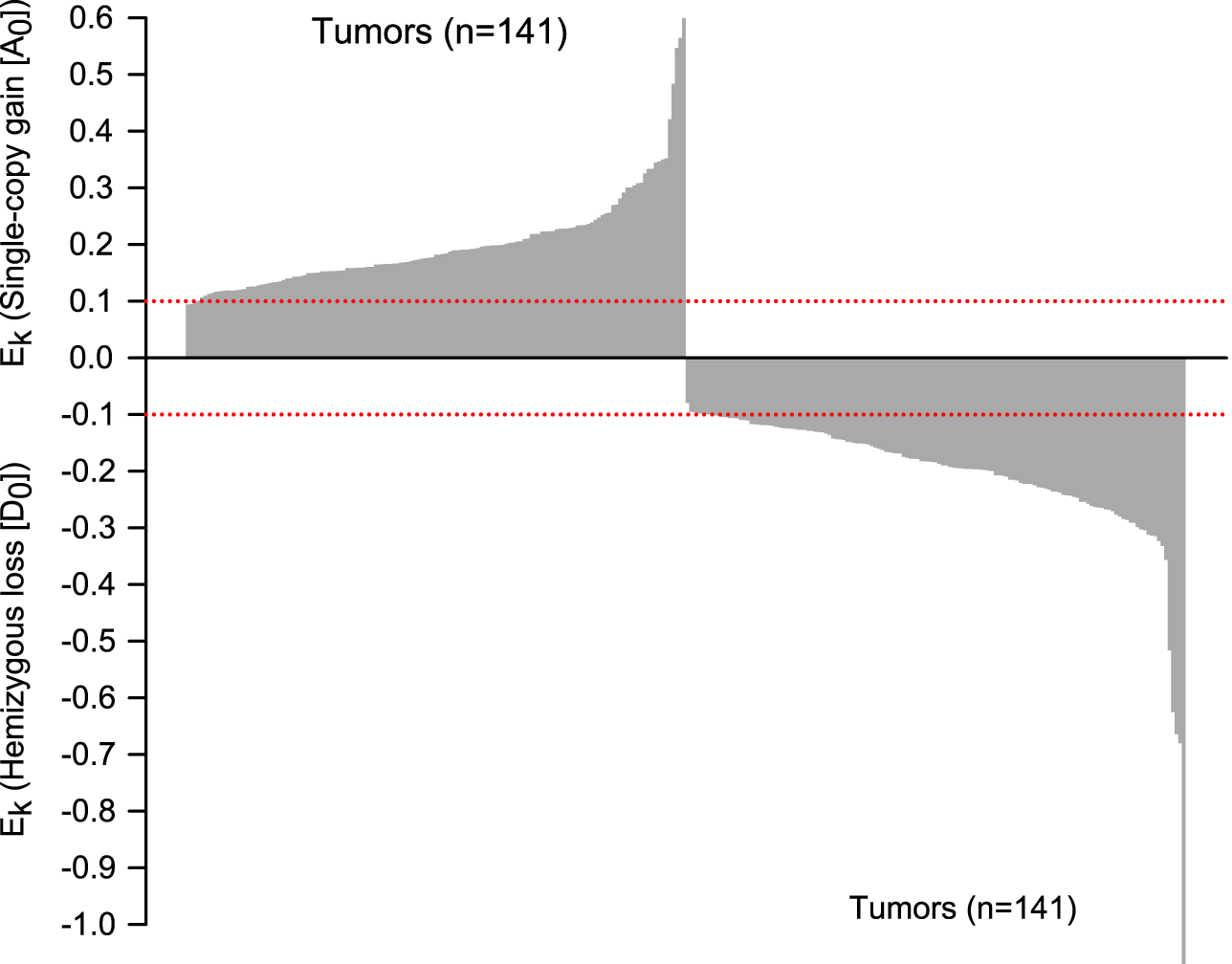

Supplement: Figure S10 — Difference between symmetric global threshold and Ek from the individual tumor noise model. Here, the Ek value for A0 and D0 (single-copy gains and losses respectively) are shown for all 141 tumors of the glioma dataset. This indicates that in the majority of tumors, the detector for single-copy events in RAE is more stringent than was the original log2 global threshold used by the original study (red dotted lines). This is responsible for the global reduction in alteration frequencies in the RAE analysis. (0.26 MB TIF) [file pone.0003179.s016.tif]
